# Microenvironmental G protein‐coupled estrogen receptor‐mediated glutamine metabolic coupling between cancer‐associated fibroblasts and triple‐negative breast cancer cells governs tumour progression

**DOI:** 10.1002/ctm2.70131

**Published:** 2024-12-17

**Authors:** Chongwu He, Meixi Peng, Xiaoqiang Zeng, Hanzhi Dong, Zhengkui Sun, Jiawei Xu, Manran Liu, Liyan Liu, Yanxiao Huang, Zhiqiang Peng, Yu‐An Qiu, Chunling Jiang, Bin Xu, Tenghua Yu

**Affiliations:** ^1^ Department of Breast Surgery, Jiangxi Cancer Hospital, The Second Affiliated Hospital of Nanchang Medical College, Jiangxi Clinical Research Center for Cancer, JXHC Key Laboratory of Tumor Microenvironment and Immunoregulation Jiangxi Key Laboratory of Tumour Metastasis of Jiangxi Health Commission Nanchang China; ^2^ Department of Radiological Medicine, School of Basic Medical Sciences Chongqing Medical University Chongqing China; ^3^ Jiangxi Medical College Nanchang University Nanchang China; ^4^ Department of Oncology The First Affiliated Hospital of Nanchang University Nanchang China; ^5^ Key Laboratory of Laboratory Medical Diagnostics, Chinese Ministry of Education Chongqing Medical University Chongqing China; ^6^ Department of Pharmacy, Jiangxi Cancer Hospital, The Second Affiliated Hospital of Nanchang Medical College Jiangxi Clinical Research Center for Cancer Nanchang China; ^7^ Department of Lymphohematology, Jiangxi Cancer Hospital, The Second Affiliated Hospital of Nanchang Medical College Jiangxi Clinical Research Center for Cancer Nanchang China; ^8^ Department of Critical Care Medicine, Jiangxi Cancer Hospital, The Second Affiliated Hospital of Nanchang Medical College Jiangxi Clinical Research Center for Cancer Nanchang China; ^9^ Department of Radiation Oncology, Jiangxi Cancer Hospital, The Second Affiliated Hospital of Nanchang Medical College, Key Laboratory of Personalized Diagnosis and Treatment of Nasopharyngeal Carcinoma Medical College of Nanchang University Nanchang China; ^10^ Jiangxi Health Committee Key (JHCK) Laboratory of Tumor Metastasis Jiangxi Cancer Hospital Nanchang China

**Keywords:** CAFs, glutamine metabolism, GPER, TNBC, tumour progression

## Abstract

**Background:**

Triple‐negative breast cancer (TNBC) is a particularly aggressive type of breast cancer, known for its lack of effective treatments and unfavorable prognosis. The G protein‐coupled estrogen receptor (GPER), a novel estrogen receptor, is linked to increased malignancy in various cancers. However, its involvement in the metabolic regulation of cancer‐associated fibroblasts (CAFs), a key component in the tumour microenvironment, remains largely unexplored. This study investigates how GPER influences the metabolic interaction between CAFs and TNBC cells, aiming to identify potential therapeutic targets.

**Methods:**

The co‐culture system is performed to examine the interaction between CAFs and TNBC cells, with a focus on GPER‐mediated glutamine production and release by CAFs and its subsequent uptake and utilization by TNBC cells. The definite roles of microenvironmental GPER/cAMP/PKA/CREB signalling in regulating the expression of glutamine synthetase (GLUL) and lactate dehydrogenase B (LDHB) are further investigated.

**Results:**

Our findings reveal that estrogen‐activated GPER in CAFs significantly upregulates the expression of GLUL and LDHB, leading to increased glutamine production. This glutamine is then secreted into the extracellular matrix and absorbed by TNBC cells, enhancing their viability, motility, and chemoresistance both in vitro and in vivo. TNBC cells further metabolize the glutamine through the glutamine transporter (ASCT2) and glutaminase (GLS1) axes, which, in turn, promote mitochondrial activity and tumour progression.

**Conclusions:**

The study identifies GPER as a critical mediator of metabolic coupling between CAFs and TNBC cells, primarily through glutamine metabolism. Targeting the estrogen/GPER/glutamine signalling axis in CAFs offers a promising therapeutic strategy to inhibit TNBC progression and improve patient outcomes. This novel insight into the tumour microenvironment highlights the potential of metabolic interventions in treating TNBC.

**Key points:**

1. Estrogen‐activated GPER in CAFs enhances GLUL and LDHB expression via the cAMP/PKA/CREB signalling, facilitating glutamine production and utilization.
2. Microenvironmental GPER‐induced glutamine serves as a crucial mediator of metabolic coupling between CAFs and TNBC cells, boosting tumour progression by enhancing mitochondrial function.
3. Targeting the glutamine metabolic coupling triggered by estrogen/GPER/GLUL signalling in CAFs is a promising therapeutic strategy for TNBC treatment.

## Graphical abstract

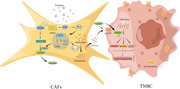

## 1 INTRODUCTION

Breast cancer is the most widespread malignancy and the top cause of cancer‐related mortality among women globally. In 2023, it is to account for 31% of all female cancer diagnoses and lead to 43 170 deaths in the United States.^1^ Triple‐negative breast cancer (TNBC), which lacks expression of classically estrogen receptor (ER) and progesterone receptor (PR) and human epidermal growth factor receptor‐2 (HER‐2) amplification, makes up 15–20% of all breast cancer cases. Its high recurrence and metastatic rates make it one of the most challenging subtypes to manage.^2^, ^3^, ^4^ Over the past decades, the traditional tumour cell‐centric perspective of cancer has evolved with an increased understanding of the tumour microenvironment (TME) and its critical role in malignant phenotype, including cancer cell proliferation, invasion, angiogenesis, and metastasis.^5^ Cancer‐associated fibroblasts (CAFs), as activated fibroblasts, are abundant in the breast TME. They promote cancer progression through direct interactions, paracrine signalling, immune modulation, and remodelling of the extracellular matrix (ECM).^6^ CAFs are now seen as active drivers of tumour progression, not merely passive bystanders. This underscores the importance of further investigating the mechanisms of interaction between TNBC cells and CAFs.

Glutamine (Gln) is a vital nutrient for cancer cells, serving as a carbon and primary nitrogen donor to support proliferating cells by providing energy and generating biosynthetic intermediates, such as amino acids and nucleotides.^7^, ^8^ It is well established that aerobic glycolysis redirects the metabolic flux of glucose to secretory lactate in cancer cells, resulting in a deficit of carbon sources from glucose for the TCA cycle and necessitating alternative sources for cell survival. Consequently, glutamine, second only to glucose, serves as a key carbon source fueling TCA cycle metabolism, thereby sustaining cancer cell survival in breast cancer and cervical cancer.^9^, ^10^, ^11^ Furthermore, the serum of TNBC patients was characterized by higher levels of glutamine compared with the healthy population, underscoring its potentially significant role in tumour progression.^12^ However, before sufficient neovascularization is established within tumours, vasogenic glutamine remains limited for cancer cells, raising questions about how these cells obtain adequate glutamine to sustain growth, metastasis, and biosynthesis within the nutrient‐deprived TME.

Estrogenic effects are largely mediated by nuclear receptors ERα and ERβ, which function as transcription factors to regulate gene expression, driving processes like cell cycle progression, migration, and survival.^13^ A significant amount of research has shown that G protein‐coupled estrogen receptor (GPER) plays a role in mediating estrogen's effects across various normal and cancerous cell types, including CAFs,^14^ cancer cells,^15^ and immune cells.^16^ Beyond estrogen, selective ER modulators like tamoxifen (TAM) and the pure ER down‐regulator fulvestrant (FUL), commonly used in ER+ breast cancer, also have a high affinity for GPER, mimicking estrogen's effects in breast cancer. This phenomenon may limit the clinical efficacy of TAM and FUL in ER+ breast cancer patients.^17^ Moreover, prior studies have demonstrated a strong correlation between GPER expression in breast cancer tissues and unfavourable clinicopathological features, such as increased tumour size and the presence of distant metastasis.^17^, ^18^ However, limited research has explored the impact of GPER in CAFs on TNBC progression, specifically regarding tumour cell invasion, metastasis, or clinical drug resistance, particularly through the regulation of glutamine metabolism.

GPER exhibits diverse intracellular localizations. Instead of remaining inactive, it may act as a shuttle under cellular conditions, moving between the membrane, nucleus, and various subcellular compartments like the endoplasmic reticulum, Golgi apparatus, and ribosomes.^19^ Interestingly, the reciprocal interaction between CAFs and tumour cells may be influenced by stromal GPER translocation, facilitating their “cross‐talk”. Our previous studies were the first to reveal that breast tumour cells induce GPER translocation to the cytoplasm in CAFs, which enhances multidrug resistance in tumour cells via glucose metabolic coupling, especially in TNBC.^20^ Moreover, breast cancer individuals with predominant stromal cytoplasmic GPER expressing predict a poor prognosis.^20^ However, the mechanism by which microenvironmental GPER triggers TNBC progression remains unclear and warrants further investigation.

This study reveals that activated cytoplasmic GPER in CAFs upregulated GLUL and LDHB expression via the cAMP/PKA/CREB signalling pathway, facilitating glutamine feeding to TNBC cells through lactate secretion. Enhanced glutamine metabolism in tumour cells subsequently amplifies their malignant potential. These findings suggest that targeting microenvironmental GPER in CAFs could represent a promising therapeutic strategy for TNBC treatment.

## 2 MATERIALS AND METHODS

### 2.1 Clinical sample

Tissue samples, including primary tumours (T) and adjacent non‐tumorous (N) areas, were gathered from 130 TNBC patients at Jiangxi Cancer Hospital (Nanchang, China). Two pathologists independently verified the tumour and normal tissue pairs. Tissue specimens were collected from July 2019 to September 2020, with informed consent from all participants. The study received approval from the Ethics Committee of Jiangxi Cancer Hospital, and all participants consented to the study and its publication.

### 2.2 Cell culture

Human GPER‐negative TNBC cells (including BT549 and MDA‐MB‐231) were obtained from the American Type Culture Collection. CAFs and normal fibroblasts (NFs) were isolated from breast tumour specimens and adjacent normal tissues. The expression of CAFs specific biomarkers (α‐SMA and FAP) and fibroblasts biomarker (FN) was confirmed by immunofluorescence in our previous studies.^21^, ^22^ Immortalized CAFs and NFs (immortalized using pBABE‐hygro‐hTERT), which have been described by our team previously.^21^, ^22^ The detailed steps are provided in supplementary materials and methods. All media and culture conditions required for the cells are provided in the cell culture section of supplementary materials and methods.

### 2.3 Reagents

E2, the specific GPER antagonist G15, adenylate cyclase inhibitor MDL‐12330, PKA antagonist H‐89, astramembrangenin, epirubicin (EPI), mitomycin C and lactate transporter inhibitor quercetin (QUE) were obtained from MedChemExpress (Monmouth Junction). The concentrations of E2, G15, MDL‐12330, and H‐89 were selected based on our previous research.^20^ All antibodies used in this study are listed in the reagents section of supplementary materials and methods.

NFs or CAFs were grown to approximately 70% confluence in a 10% fetal bovine serum (FBS) medium, then starved for 12 h in an FBS‐free medium. Subsequently, they were treated with the indicated breast cancer cell conditioned medium (CM), with or without indicated reagents (e.g., E2 (100 nM), G15 (100 nM), MDL‐12330 (20 µM), H‐89 (30 µM), EPI (1.2 µg/mL)).

### 2.4 Chromatin immunoprecipitation

CAFs were plated at a concentration of 1 × 10⁶ cells in a 10 mm dish. Following a 24 h incubation with CM from BT549, the cells were treated with formaldehyde at 4°C for 12 min, followed by quenching with glycine (.125 mol/L). Chromatin was fragmented into small pieces using sonication. The target protein was immunoprecipitated using anti‐CREB antibody and protein G beads. Protein digestion was carried out using proteinase K at 45°C for 50 min. The DNA bound to the target protein was purified and collected using HSYBR qPCR Mix (Zomanbio). The following primers were used to amplify the potential CREB binding region in the GLUL promoter: forward 5′‐TCAATCTCTTCTGCATGTGCTAT‐3′, reverse 5′‐ACTTAGCCACATGAAGCCTGT‐3′. The following primers were used to amplify the potential CREB binding region in the LDHB promoter: forward 5′‐CAGGGACAAAGCCTCATTGG‐3′ and reverse 5′‐AACCTGTTTCAACAAACACGCAA‐3′. Each experiment was independently conducted at least three times.

### 2.5 Measurement of cell mitochondrial activity

In the transwell coculture system between CAFs or NFs and breast cancer cells, CAFs were treated with or without E2 and G15. The mitochondrial function was evaluated using the enhanced mitochondrial membrane potential assay kit with JC‐1 (Beyotime) according to the manufacturer's protocol and detected using a Flow cytometer (BD FACSAria Fusion, BD).

### 2.6 Immunohischemistry

Tumorous and normal tissues were fixed with 4% paraformaldehyde and then sectioned into 4 µm thickness. Immunohischemistry (IHC) was performed following the manufacturer's protocols. Tissue sections were incubated with the specified antibodies overnight at 4°C. Subsequently, the sections were sequentially incubated with polyperoxidase‐anti‐rabbit IgG (ZSBiO) for 30 min at 37°C, followed by diaminobenizidine. Images were captured and evaluated by Image‐Pro 10.0 software (Media Cybernetics) and scored by mean optical density (density/area). GPER, GLUL, ASCT2, and GLS1 staining proportions were scored into five intensities: 0, no staining; 1 +, 1–25%; 2 +, 26–50%; 3 +, 51–75%, and 76–100%, while the grades of immunostaining intensity were 0: negative, 1: weak, 2: moderate, and 3, strong.

### 2.7 Orthotopic xenografts and lung metastasis analysis

The animal experiments received approval from the animal care ethics committees at Chongqing Medical University. MDA‐MB‐231 cells (1 × 10^6^) mixed with either control CAFs (CAFs/Ctrl) or engineered CAFs (1 × 10^6^) in 200 µL of PBS: Matrigel at a 1:1 ratio were subcutaneously injected into 4‐week‐old female nude mice. The following experimental procedures were performed according to our previous publication.^23^

### 2.8 Statistical analysis

Statistical significance was conducted with SPSS 22.0 software. All experiments were performed in triplicate, with results presented as the mean ± SD. Continuous variables between two groups were analyzed using the independent Student's *t*‐test while multiple group comparisons were conducted using ANOVA followed by the Student–Newman–Keuls multiple comparison test. The correlation between cytoplasmic GPER expression and GLUL expression was estimated by *P*earson's correlation. A *p*‐value < .05 was considered statistically significant.

## 3 RESULTS

### 3.1 GPER mediates the biosynthesis and secretion of glutamine in CAFs

**FIGURE 1:**
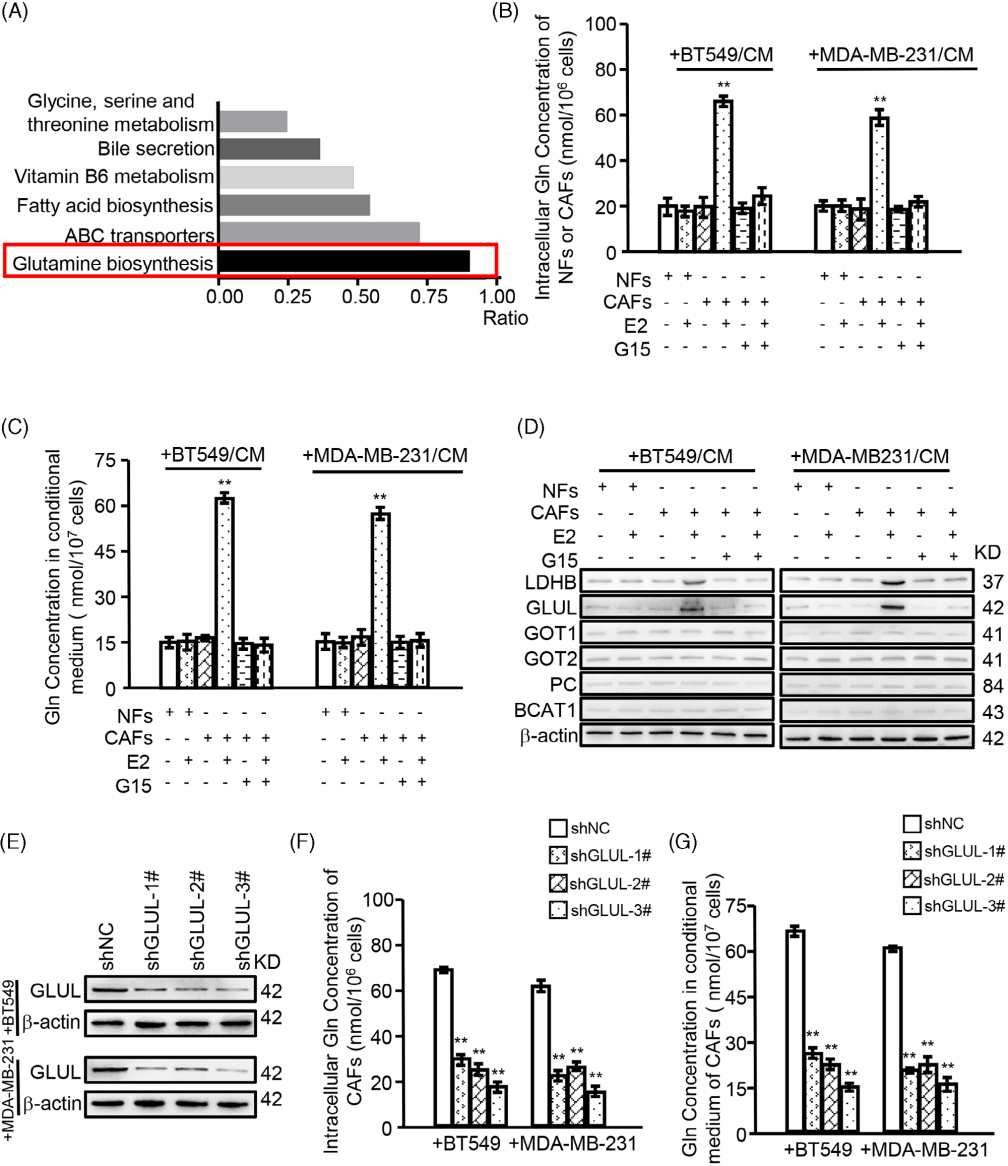
GPER mediates the biosynthesis and secretion of glutamine in CAFs. NFs or CAFs were cultured with CM from BT549 or MDA‐MB‐231, and treated with or without indicated reagents (e.g., E2 (100 nM), G15 (100 nM)) for 12 h. (A) Metabonomics analysis identified the altered metabolites between GPER‐activated CAFs (treated with E2) and GPER‐inactivated CAFs (treated without E2). Relative metabolic signalling pathways were enriched using KEGG analysis. The ratio represents the number of altered metabolites to the total number of metabolites in the pathway. (B, C) The production of glutamine in NFs and CAFs (B) and the concentration of glutamine in CM (C) are shown (*n* = 3). (D) Western blotting was used to determine the expression of indicated glutamine synthesis‐related genes. (E) The efficiencies of GLUL silence in the indicated cells were detected by western blotting. (F, G) GLUL‐silenced CAFs were cultured with CM from BT549 or MDA‐MB‐231 in the presence of E2. The production of glutamine in CAFs (F) and the concentration of glutamine in CM (G) are shown (*n* = 3). Data represent mean ± SD. *p*‐values were calculated using a student *t‐*test. The significance of multiple group comparisons was analyzed by one‐way ANOVA. ** *p *< .01.

Our previous study demonstrated that cytoplasmic GPER in CAFs reduced breast cancer cell drug sensitivities to TAM, Herceptin, or epirubicin by providing tumour cells with lactate and pyruvate.^20^ Nonetheless, the precise mechanism remained elusive. To further investigate the regulatory role of GPER in CAFs on TNBC progression, a metabonomics assay was conducted on CAFs with or without GPER activation. That activation of GPER led to significant alterations in metabolite expression, with 98 metabolites upregulated and 123 downregulated (*p *< .05). KEGG analysis of these altered metabolites indicated an enrichment in “glutamine biosynthesis” (Figure 1A). Additionally, glutamine has been reported as essential for sustaining breast cancer growth and malignancy.^9^, ^24^ These results imply that microenvironmental GPER may regulate glutamine biosynthesis.

As GPER can be activated in CAFs co‐cultured with TNBC cells and treated with E2 (an agonist of GPER and ERα) simultaneously,^20^ we first analyzed the expression levels of ERα, ERβ, and GPER in the indicated cell lines. As shown in Figure S1A, compared with MCF7 (ER‐positive breast cancer cell line) and MDA‐MB‐468 (GPER‐positive TNBC cell line), ERα, ERβ, and GPER were significantly either very low or not expressed in NFs, BT549, and MDA‐MB‐231 cells. CAFs show negative expression of ERα and ERβ, but high expression of GPER. Next, we explored whether activated GPER in CAFs regulated glutamine biosynthesis. Both intracellular and extracellular glutamine production were enhanced when GPER activated and inhibited when G15 existent in CAFs, not in NFs (Figure 1B,C). Notably, the knockdown of GPER in BT549 and MDA‐MB‐231 did not affect the glutamine level of CAFs in the co‐culture system (Figure S1B,D). Also, the mRNA and protein expression levels of glutamine biosynthesis‐related enzymes (Figure S1E, red‐labelled) were assessed, showing that LDHB and GLUL were up‐regulated when E2 activated GPER and downregulated when G15 antagonized GPER (Figure 1D; Figure S1F,G). Moreover, knocking down GLUL in GPER‐activated CAFs resulted in decreased glutamine levels in both intracellular and conditional medium (Figure 1E–G). However, there was no significant change in glutamine levels when LDHB was knocked down (Figure S2A–C). These findings indicate that GPER enhances glutamine biosynthesis and secretion in CAFs primarily by regulating GLUL expression.

As lactate in the TME may promote glutamine uptake of cancer cells,^25^ and LDHB is a key rate‐limiting enzyme in lactate biosynthesis, it was speculated whether the enhanced LDHB in GPER‐activated CAFs promoted glutamine uptake in TNBC cells. In the co‐culture system using transwells, the glutamine content in cancer cells increased with E2 treatment and decreased as GPER‐antagonized (Figure S2D). While there was no significant change in glutamine content in CAFs when knockdown LDHB (Figure S2E). Additionally, an enhanced glutamine content in the medium (Figure S2F) and a decreased glutamine content in cancer cells co‐cultured with LDHB knockdown CAFs (Figure S2G) indicated that downregulating LDHB partially inhibited glutamine uptake by TNBC cells. Collectively, these data demonstrate that GPER mediates glutamine biosynthesis and secretion in CAFs co‐cultured with TNBC cells by regulating GLUL expression and enhances glutamine uptake in tumour cells by regulating LDHB expression.

### 3.2 Cytoplasmic GPER‐related glutamine is high in clinical stromal fibroblasts, and correlated with poor prognostic features

**FIGURE 2:**
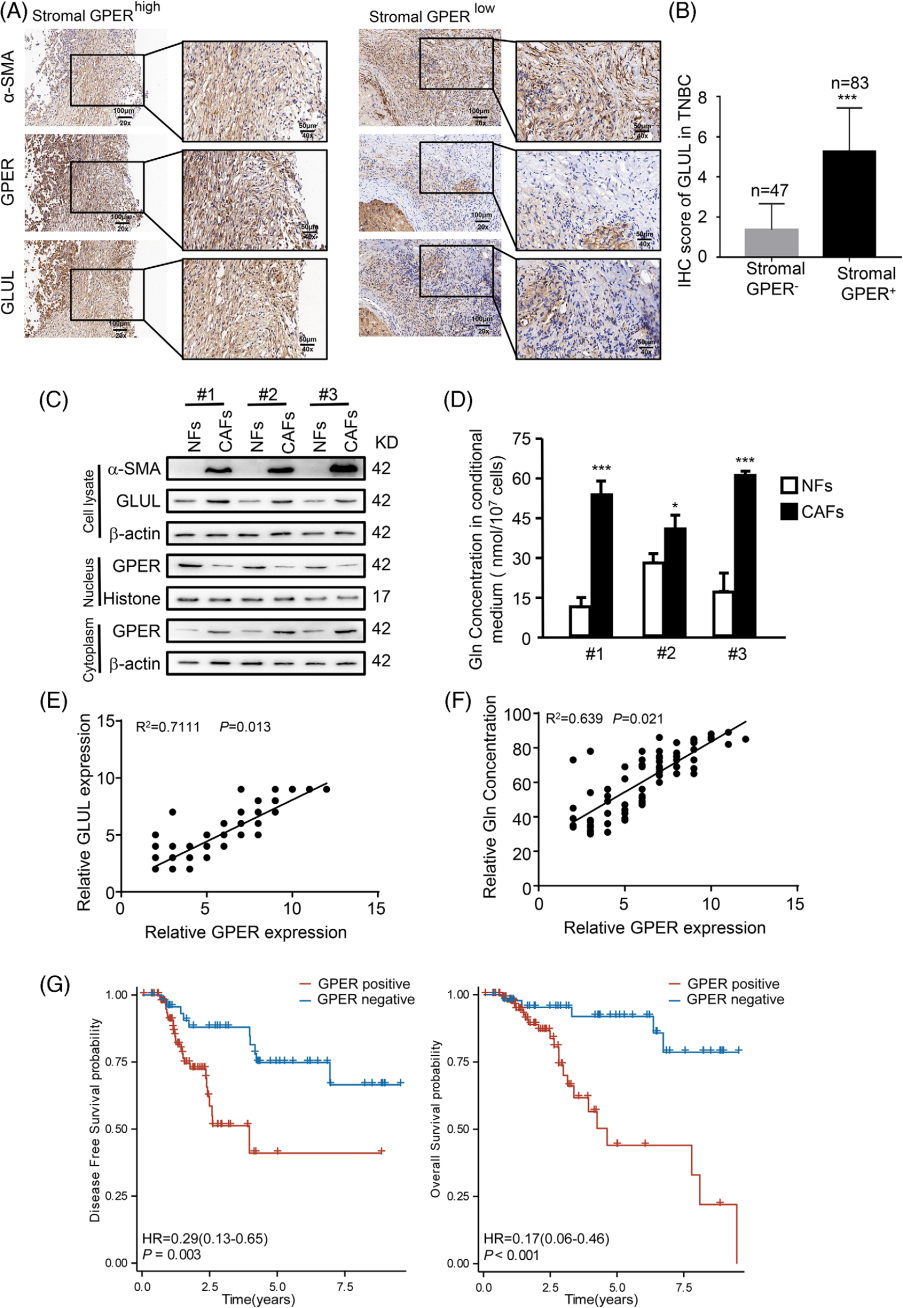
Cytoplasmic GPER‐related glutamine is high in clinical stromal fibroblasts and correlated with poor prognostic features. (A) The expression of α‐SMA, GPER, and GLUL in stromal fibroblasts of TNBC tissues was determined by IHC. (B) Characteristics expression of cytoplasmic GPER and GLUL of stromal fibroblasts in 130 TNBC patients. (C) Protein levels of cytoplasmic GPER, GLUL, and α‐SMA were detected by western blotting. (D) Glutamine levels in paired primary NFs and CAFs from patients are shown (*n* = 3). (E, F) The correlation between GPER expression and GLUL expression (E) and glutamine levels (F) are shown. (G) The correlation between cytoplasmic GPER in stromal fibroblasts and the prognosis (disease‐free survival and overall survival) of patients with TNBC is presented. Scale bars, 50 µm (magnification, ×400). Data represent mean ± SD. *p*‐values were calculated using a student *t*‐test. * *p *< .05, *** *p *< .001.

To further investigate the role of cytoplasmic GPER‐related glutamine in the malignant progression of TNBC, 130 primary TNBC tumour tissues and their paired normal mammary tissues were collected. Interestingly, 83 TNBC tumour samples (63.8%) were identified as cytoplasmic GPER‐positive in stromal fibroblasts, whereas normal mammary tissues were negative for cytoplasmic GPER in stromal fibroblasts (Figure S2H). To confirm that GPER and GLUL are coordinative expressed in CAFs, IHC experiments were performed and co‐labelled the common CAF marker α‐SMA with GPER and GLUL, respectively. The results showed that α‐SMA co‐localized with both GPER and GLUL in CAFs (Figure 2A). Moreover, GLUL expression in stromal fibroblasts was higher in the 83 patients with TNBC exhibiting cytoplasmic GPER‐positive status compared with the 47 patients with cytoplasmic GPER‐negative status (Figure 2B). Additionally, the expressions of cytoplasmic GPER and GLUL were evaluated in three paired primary NFs and CAFs isolated from the 83 TNBC patients. These primary NFs and CAFs exhibited spindle‐like morphology (data not shown), and the CAFs expressed the established biomarker α‐SMA (Figure 2C). As expected, cytoplasmic GPER expression, GLUL expression, and glutamine production were at higher levels in CAFs than in NFs (Figure 2C,D). Furthermore, cytoplasmic GPER expression positively correlated with GLUL expression and glutamine production in primary CAFs (Figure 2E,F). Importantly, high levels of cytoplasmic GPER, GLUL, and glutamine production in CAFs were associated with adverse pathological characteristics of tumours, such as large tumour size, high TNM stage, poor histology grade, and reduced survival (Table 1, Figure 2G). Collectively, these data indicate that cytoplasmic GPER‐related glutamine is elevated in CAFs from TNBC and may play a significant role in promoting TNBC development.

**TABLE 1:** The clinicopathological characteristics of 130 TNBC patients.

	Cytoplasmic GPER expression		
Characteristics	Positive	Negative	*p*‐value
Total cases, *n* (%)	83 (63.8)	47 (36.2)	
Age, median (IQR)	52 (45, 59)	55 (47.5, 64)	.217^a^
Tumour size stage, *n* (%)			<.001^b^
T1	6 (4.6%)	22 (16.9%)	
T2	63 (48.5%)	22 (16.9%)	
T3	10 (7.7%)	2 (1.5%)	
T4	4 (3.1%)	1 (.8%)	
Nodal stage, *n* (%)			.566^b^
N0	48 (36.9%)	33 (25.4%)	
N1	20 (15.4%)	8 (6.2%)	
N2	9 (6.9%)	4 (3.1%)	
N3	6 (4.6%)	2 (1.5%)	
TNM stage, *n* (%)			<.001^c^
I	0 (0%)	19 (14.6%)	
II	63 (48.5%)	20 (15.4%)	
III	20 (15.4%)	8 (6.2%)	
Histology grade, *n* (%)			<.001^b^
I	0 (0%)	6 (4.6%)	
II	2 (1.5%)	11 (8.5%)	
III	81 (62.3%)	30 (23.1%)	

Abbreviations: IQR, interquartile range; N, node; T, tumour; TNM, tumour, node, and metastasis stage.

^a^ Wilcoxon rank sum test;

^b^ Yate's correct test;

^c^ Chi‐square test

### 3.3 GPER‐induced glutamine in CAFs fuels TNBC malignant potential

**FIGURE 3:**
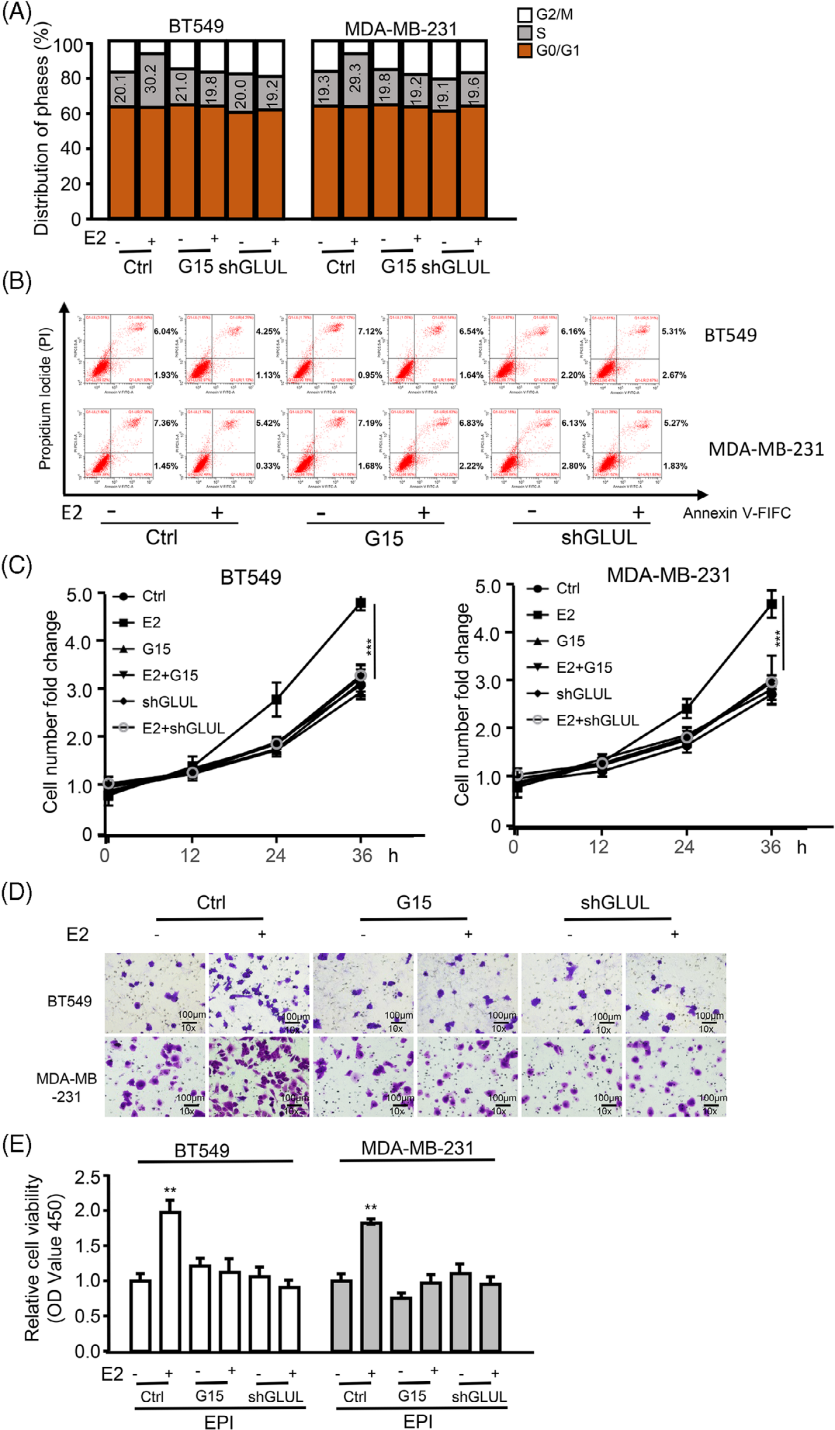
GPER‐induced glutamine in CAFs fuels TNBC malignant potential. (A–E) BT549 and MDA‐MB‐231 cells were treated with indicated reagents (e.g., E2 [100 nM, 12 h], G15 [100 nM, 12 h]), or subjected to gene knockdown and co‐cultured with CAFs. (A) The S‐phase cell ratio of BT549 and MDA‐MB‐231 cells was measured by flow cytometric assay (*n* = 3). (B) Cell apoptosis of BT549 and MDA‐MB‐231 cells was determined using Annexin V‐FITC kit and flow cytometric. Early apoptotic cells are in the lower right quadrant; late‐stage apoptotic cells are in the upper right quadrant; viable cells are in the lower left quadrant (*n* = 3). (C) Cellular proliferation was assessed using the CCK8 assay (*n* = 3). (D) The invasion of BT549 and MDA‐MB‐231 cells was evaluated via transwell assay, with quantitative diagrams of invaded cells. Scale bar, 100 µm (magnification, ×100) (*n* = 3). (E) The viability of BT549 and MDA‐MB‐231 in the presence of EPI (1.2 µg/mL) was determined by the CCK8 kit (*n* = 3). Data represent mean ± SD. *p*‐values were calculated using a student *t*‐test. **p < .01, ***p < .001.

Next, the effect of GPER‐induced glutamine in CAFs on the malignant phenotype of TNBC cells was investigated. By culturing BT549 and MDA‐MB‐231 with CAFs, cell‐cycle analysis, cell apoptosis, cell proliferation, cell invasion, and drug resistance were assessed. Cell‐cycle analysis revealed an increase in the fraction of the S phase, indicating a higher proliferation rate in BT549 and MDA‐MB‐231 when CAFs were treated with E2 (Figure 3A). However, the S phase fraction ratio was restored when GLUL was knocked down or when G15 was used in E2‐stimulated CAFs (Figure 3A). Similarly, cancer cell apoptosis decreased (Figure 3B; Figure S3A) while cell proliferation (Figure 3C), cell invasion (Figure 3D; Figure S3B), and drug resistance for EPI (Figure 3E) increased with GPER activation in E2‐treated CAFs. Conversely, cell cycle, apoptosis, proliferation, invasion, and drug resistance for EPI were reversed when GPER was inactivated or GLUL was knocked down in E2‐stimulated CAFs (Figure 3A–E; Figure S3A–C). Furthermore, the S phase fraction (Figure S3D), cell proliferation (Figure S3F), invasion (Figure S3G), and drug resistance for EPI (Figure S3H) were enhanced while cell apoptosis was decreased (Figure S3E) in TNBC cells cultured with high level (4 mM) of glutamine compared with low level (1 mM). These results illustrate the critical role of GPER‐induced glutamine in CAFs in promoting TNBC cell proliferation, survival, invasion, and chemoresistance.

### 3.4 GPER mediates GLUL and LDHB expression via cAMP/PKA/CREB signalling pathway in CAFs

**FIGURE 4:**
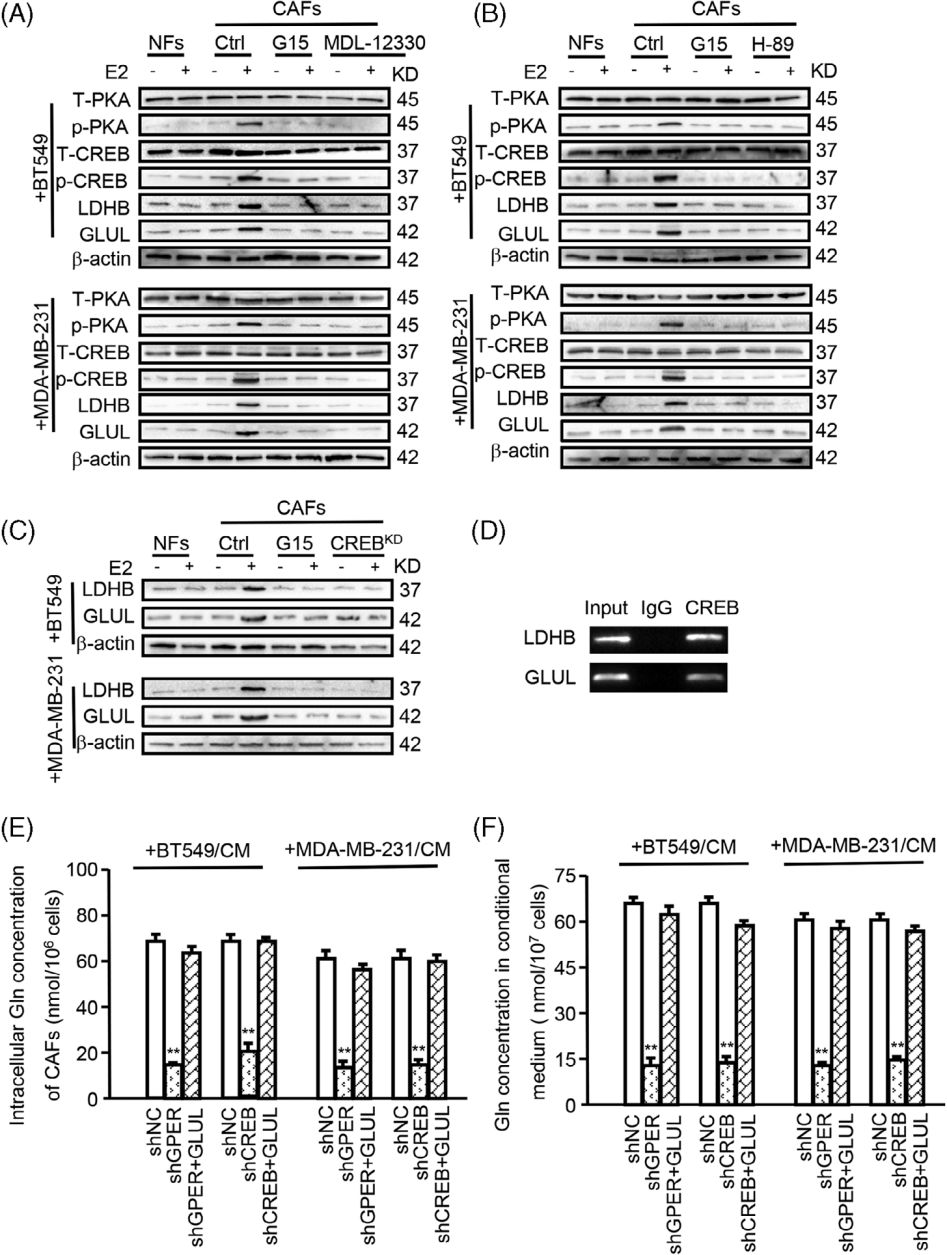
GPER mediates GLUL and LDHB expression via the cAMP/PKA/CREB signalling pathway in CAFs. (A–F) NFs or CAFs were cultured with CM from BT549 or MDA‐MB‐231 and treated with indicated reagents (e.g., E2 (100 nM, 12 h), G15 (100 nM, 12 h), MDL‐12330 (20 µM, 12 h), H‐89 (30 µM, 24 h)), or subjected to gene knockdown. (A–C) Levels of T‐PKA, p‐PKA, T‐CREB, p‐CREB, LDHB, or GLUL were measured by western blotting. (D) The transcriptional activity of GLUL and LDHB genes regulated by CREB was evaluated using a chromatin immunoprecipitation assay. (E, F) Ectopic CREB was transfected into GPER‐knockdown CAFs, and intracellular glutamine concentration in CAFs (E) and in conditional medium (F) was determined (*n* = 3). Data represent mean ± SD. *p*‐values were calculated using a student *t*‐test. ** *p *< .01.

To further elucidate the mechanism underlying microenvironmental GPER‐induced glutamine change, several key observations were made. GPER activation in E2‐treated CAFs led to increased cAMP production (Figure S4A), phosphorylated PKA (p‐PKA) expression, phosphorylated CREB (p‐CREB) expression, and elevated levels of GLUL and LDHB expression as GPER‐activated in E2‐treated CAFs (Figure 4A). Conversely, CAFs treated with G15 exhibited decreased cAMP production (Figure S4A), p‐PKA expression, p‐CREB expression, GLUL, and LDHB levels (Figure 4A). Inhibition of cAMP by MDL‐12330 also reduced GPER‐induced p‐PKA, p‐CREB, GLUL, and LDHB expression (Figure 4A). Consistently, treatment with H‐89 led to decreased levels of GPER‐induced p‐CREB, GLUL, and LDHB in CAFs (Figure 4B). Moreover, CREB knockdown suppressed GPER‐induced GLUL and LDHB (Figure S4B; Figure 4C) while activation of CREB with astramembrangenin (a CREB activator) enhanced GLUL and LDHB levels in CAFs (Figure S4C). Notably, CREB was identified as a potential transcription factor for GLUL and LDHB using the hTFtarget database (Figure S4D). Chromatin immunoprecipitation assays demonstrated that CREB could bind to the GLUL and LDHB promoters (Figure 4D), and dual‐luciferase assays confirmed that the CREB enhanced GLUL and LDHB transcription (Figure S4E). Knockdown of GPER or CREB significantly decreased glutamine production in CAFs and the conditional medium (Figure 4E,F). Transfection of GLUL into GPER or CREB knockdown CAFs restored glutamine levels in cells and conditional medium (Figure 4E,F). These data indicate that GPER regulates GLUL and LDHB expression via the cAMP/PKA/CREB signalling pathway in CAFs.

### 3.5 Microenvironmental GPER‐related glutamine enhanced glutamine metabolism in TNBC cells

**FIGURE 5:**
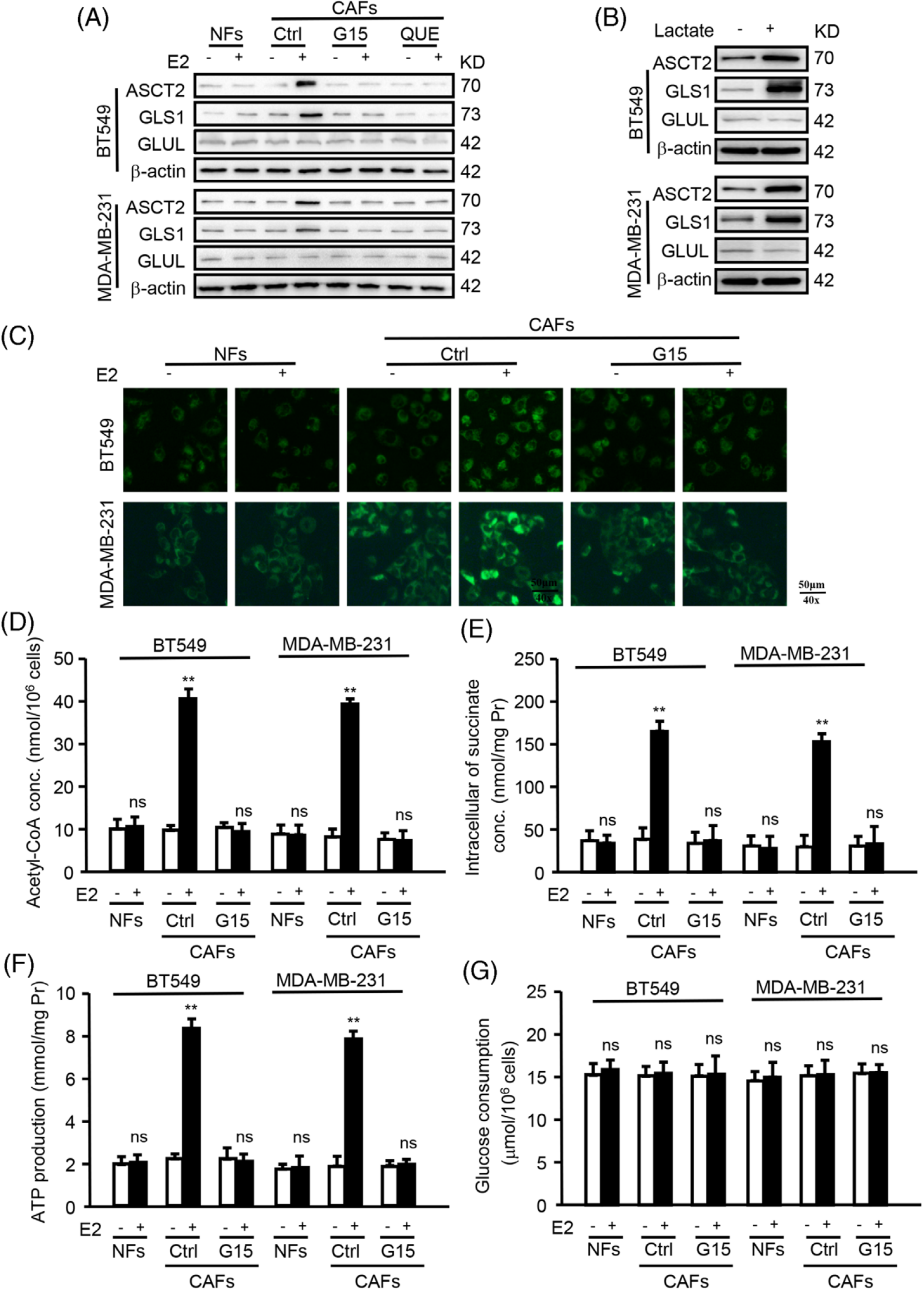
Microenvironmental GPER‐related glutamine enhanced glutamine metabolism in TNBC cells. (A) The expression of ASCT2, GLS1, GLUL of BT549 and MDA‐MB‐231 co‐cultured with E2‐treated CAFs was detected by western blotting. (B) The expression of ASCT2, GLS1, and GLUL in BT549 and MDA‐MB‐231 treated with or without lactate (20 mM, 24 h) was measured by western blotting. (C–G) BT549 or MDA‐MB‐231 cells co‐cultured with CAFs. Mitochondrial activity (green) (C), ATP production (D), acetyl‐CoA concentration (E), succinate concentration (F), and glucose consumption (G) in BT549 and MDA‐MB‐231 were detected using corresponding reagent kits (*n* = 3). Scale bars, 50 µm (magnification, ×400). Data represent mean ± SD. *p*‐values were calculated using a student *t*‐test. **p < .01; ns, no significance.

Given that GPER in CAFs enhances glutamine uptake in cancer cells by regulating LDHB expression, the underlying molecular mechanism of LDHB‐regulated lactate on glutamine absorption was investigated. Notably, the expression levels of ASCT2 and GLS1, the primary enzymes involved in glutamine uptake and metabolism, were elevated in TNBC cells when GPER in CAFs was activated and reduced when GPER was inactivated or treated with QUE (Figure 5A). Conversely, GLUL expression in tumour cells remained unchanged (Figure 5A). Additionally, lactate treatment increased the expression of ASCT2 and GLS1, but not GLUL, in cancer cells (Figure 5B). These results indicate that the GPER‐induced lactate microenvironment from CAFs, rather than the tumour cells themselves, plays a pivotal role in the transport and metabolism of glutamine in TNBC cells.

Glutamine is reported to serve as an anaplerosis substrate, fueling the TCA cycle for energy generation and providing nitrogen for protein synthesis.^26^ Additionally, mitochondrial oxidative phosphorylation (OXPHOS) is a key regulator of breast tumour metastasis.^27^ This study investigated whether microenvironmental GPER‐related glutamine enhances tumour progression by fueling the TCA cycle. Mitochondrial metabolism was assessed, revealing increased mitochondrial activity (Figure 5C), acetyl‐CoA concentration (Figure 5D), succinate concentration (Figure 5E), and ATP production (Figure 5F) were increased in TNBC cells when GPER activated in CAFs, which decreased when GPER was inactivated. Glucose consumption, however, remained unchanged (Figure 5G). Moreover, enhanced mitochondrial activity (Figure S5A), acetyl‐CoA concentration (Figure S5B), succinate concentration (Figure S5C), and ATP production (Figure S5D) were observed in cancer cells with high levels (4 mM) of glutamine compared with low level (1 mM) in the presence of lactate (20 mM). These results indicate that GPER in CAFs promotes the anabolic pathway and OXPHOS in tumour cells via glutamine metabolism reprogramming, independent of glucose consumption.

### 3.6 GPER/GLUL‐induced glutamine metabolic coupling in CAFs promotes TNBC progression in vivo

**FIGURE 6:**
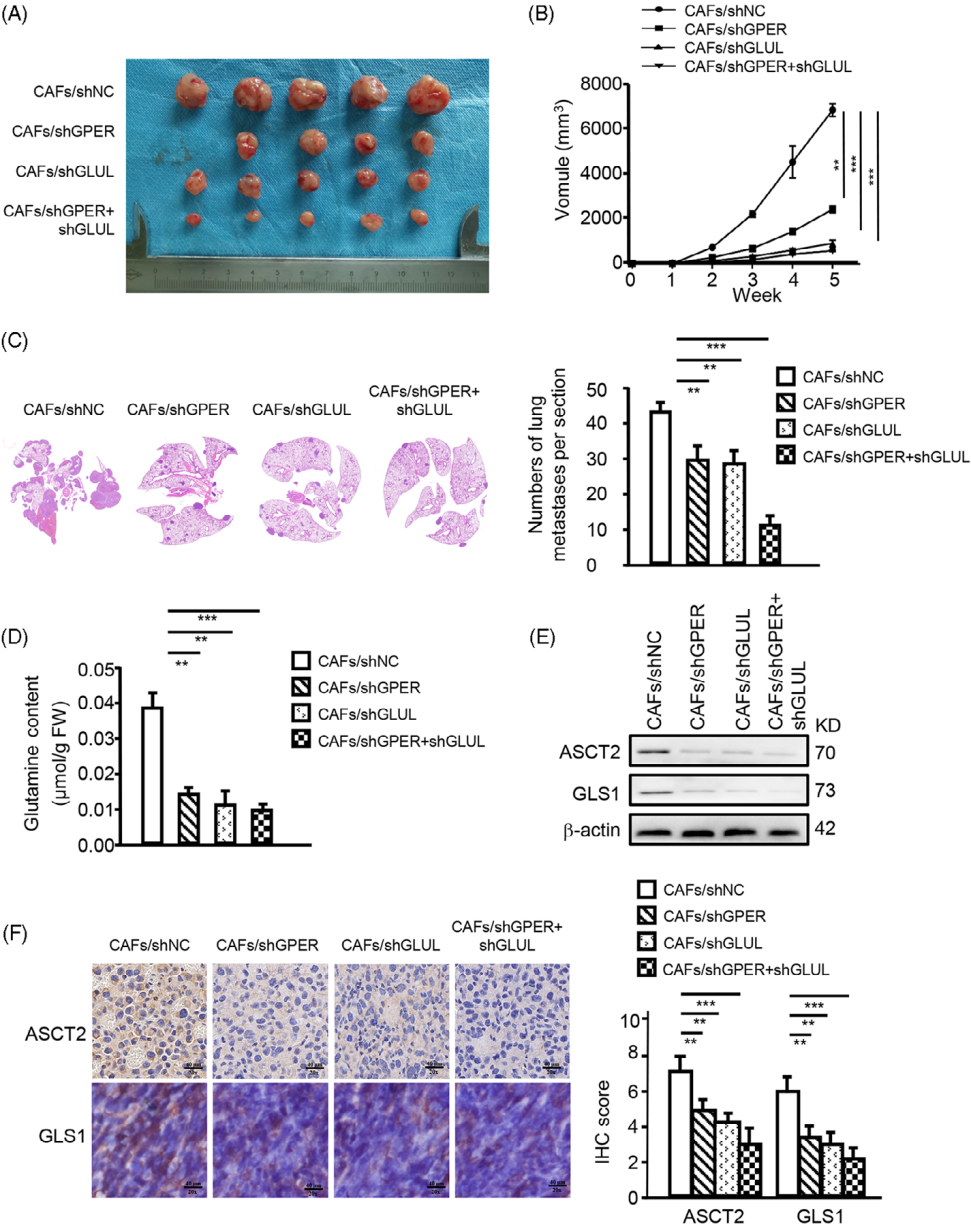
Microenvironmental GPER‐induced glutamine in CAFs promotes TNBC progression in vivo. MDA‐MB‐231 TNBC cells mixed with CAFs or engineered CAFs (CAFs/shGPER, CAFs/shGLUL, CAFs/shGPER+shGLUL) were subcutaneously transplanted into nude mice. (A) Tumour size in mice (*n* = 5 mice per group). (B) Tumour growth curve showing data from the four groups described in (A) (*n* = 5 mice per group). (C) Representative images of lung metastases were checked using H&E staining. Quantitative diagrams of metastases in mice lungs are shown (*n* = 5 mice per group). (D) Glutamine content in mice tumours, normalized by tissue weight (*n* = 5 mice per group). (E, F) Protein levels of ASCT2 and GLS1 in tumour tissues were detected by Western blotting (E) and IHC staining (*n* = 5 mice per group) (F). Representative images of ASCT2 and GLS1 examined by IHC staining are shown in (F) (*n* = 5 mice per group). Scale bar, 40 µm (magnification, ×200). Data represent mean ± SD. *p‐*values were calculated using a student *t*‐test. The significance of multiple group comparisons was analyzed by one‐way ANOVA. **p < .01; ***p < .001.

To confirm that GPER/GLUL‐induced glutamine from CAFs promotes tumour progression, MDA‐MB‐231 cells mixed with CAFs, or engineered CAFs (CAFs/shGPER, CAFs/shGLUL, CAFs/shGPER+shGLUL) were subcutaneously transplanted into nude mice. Consistent with in vitro data, mice injected with MDA‐MB‐231 and engineered CAFs developed significantly smaller tumours (Figure 6A), exhibited slower growth (Figure 6B), and had fewer lung metastases (Figure 6C) compared with those injected with a mixture of MDA‐MB‐231 and control CAFs. Notably, blocking glutamine metabolic coupling by knocking down GPER and/or GLUL in CAFs resulted in reduced glutamine production (Figure 6D) and decreased expression of ASCT2 and GLS1 in tumours (Figure 6E,F). These findings support the hypothesis that GPER in CAFs promotes TNBC progression through glutamine transfer between CAFs and cancer cells.

## 4 DISCUSSION

**FIGURE 7:**
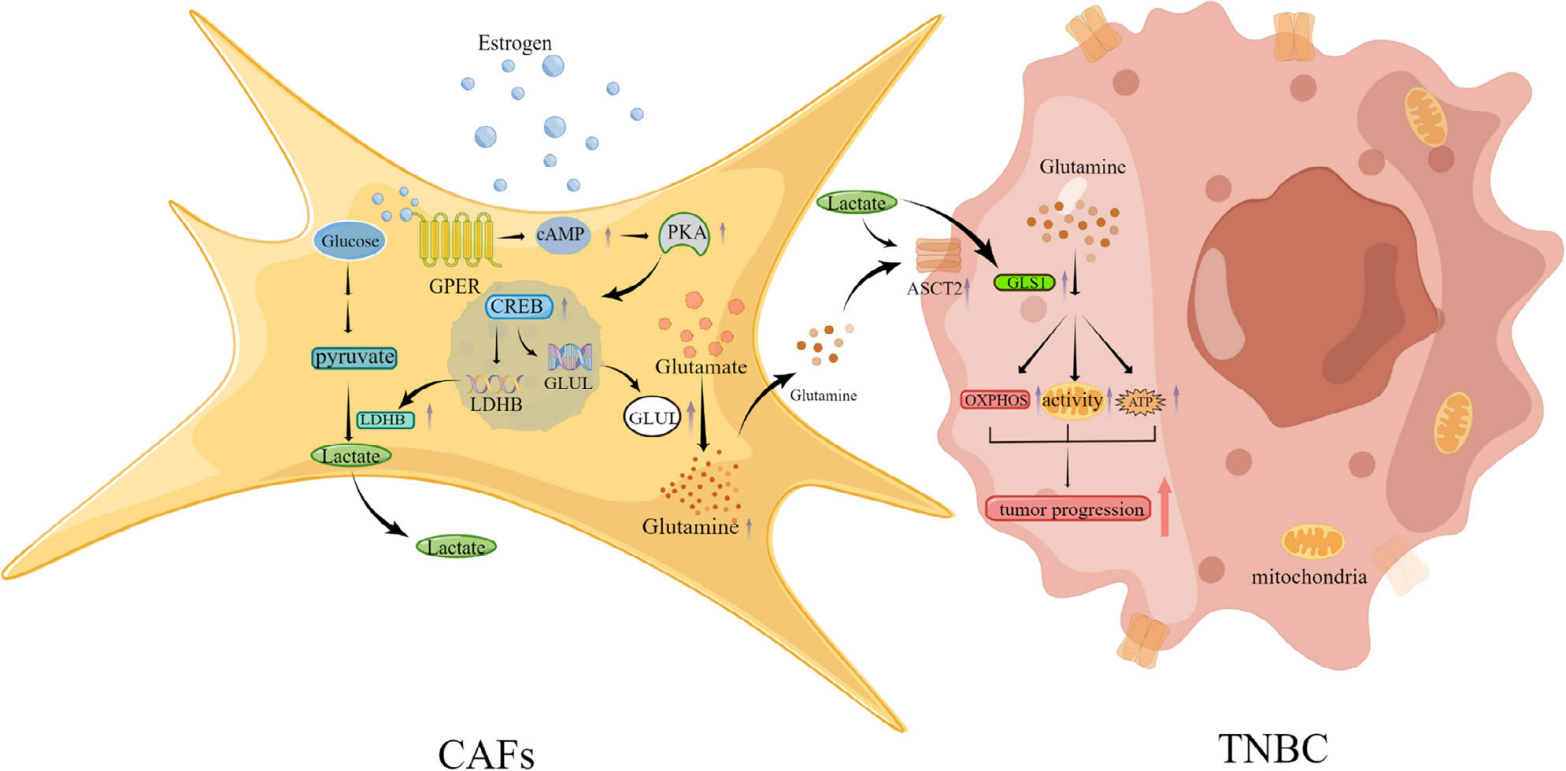
A model for CAFs governing TNBC tumour progression through microenvironmental GPER‐mediated glutamine metabolism. Cytoplasmic GPER activated by estrogen in CAFs upregulated GLUL and LDHB expression via the cAMP/PKA/CREB signalling pathway, promoting glutamine synthesis. GPER‐induced glutamine is secreted into the extracellular microenvironment, where TNBC cells uptake and metabolize it to enhance their glutamine metabolism. During this process, lactate can increase the transport of glutamine to breast cancer cells. GPER‐associated glutamine boosts the malignant potential of TNBC cells.

TNBC remains a challenging issue in the clinic, accounting for over 50% of breast cancer mortality. Estrogen is typically regarded as having minimal impact on TNBC because of the cancer's weak response to conventional hormonal therapies. However, clinical data indicate that TNBC is more prevalent in premenopausal and younger patients, suggesting a potential link between female hormones and TNBC progression. GPER has been identified as a novel estrogen receptor mediating rapid and non‐genomic estrogenic effects. This study revealed that estrogen‐activated cytoplasmic GPER in CAFs leads to glutamine synthesis and secretion into the extracellular matrix via the cAMP/PKA/CREB/GLUL pathway. Additionally, the estrogen/GPER/PKA/LDHB/lactate axis in CAFs induces tumour cells to express ASCT2 and GLS1, enhancing the absorption and utilization of GPER‐induced glutamine in TNBC. GPER‐mediated glutamine metabolic coupling in the microenvironment fuels mitochondrial activity and promotes the malignant properties of TNBC (Figure 7). This work highlights the influence of estrogen on TNBC from the perspective of the GPER‐mediated metabolic microenvironment and may offer new insight into TNBC therapy.

Glutamine is a vital nutrient for cancer cell survival, providing amido nitrogen for amino sugar and nucleotide biosynthesis and entering the TCA cycle to produce energy for cell survival.^28^ In the TME, rapidly proliferating tumour cells deplete nutrients, leading to a scarcity of glutamine, glucose, and other essential molecules.^29^, ^30^ However, the mechanisms by which cancer cells maintain glutamine levels in the TME, especially during early angiogenesis, remain unclear. This study demonstrates that GPER enhances glutamine synthesis and secretion from CAFs into the extracellular matrix, where it is absorbed by cancer cells to support their survival. Over the past decade, CAFs have been known to promote cancer progression through multiple mechanisms. For example, IL‐32 and TGFβ act as mediators facilitating cross‐talk between breast CAFs and cancer cells, promoting tumour cell proliferation and invasion.^23^ Additionally, metabolic couples, such as lactate exchange CAFs and tumour cells, play a pivotal role in tumour cell invasion.^31^ This research reveals a significant function of GPER in mediating glutamine metabolic coupling between CAFs and TNBC cells, which is crucial for tumour survival. These findings may shed light on the mechanisms underlying TNBC development, particularly in the early stages.

Microenvironmental cytoplasmic GPER regulates the biosynthesis and secretion of glutamine. In altered cellular conditions, intracellular GPER may function as a shuttle rather than remaining inactive.^32^ Our earlier studies revealed that breast cancer cells can trigger the movement of GPER from the nucleus to the cytoplasm in CAFs. Cytoplasmic GPER causes glycolytic CAFs to produce energy‐rich pyruvate and lactate.^20^ GLUL, the key enzyme catalyzing the ATP‐dependent synthesis of glutamine from glutamate and ammonia, plays a critical role in detoxifying glutamate and ammonia and maintaining acid–base homeostasis.^33^ GLUL to positively influence breast cancer cell proliferation^34^ and serve as an unfavourable prognostic marker in patients with glioblastoma multiforme and ovarian cancer.^35^, ^36^ In the stromal components, targeting stromal GLUL might offer a promising therapeutic strategy.^37^, ^38^ However, the specific regulatory mechanism of tumour glutamine metabolism through the stromal GLUL in the TNBC microenvironment, especially the close relationship among GPER, GLUL, and CAFs, still demands further exploration. IHC results in this study suggested a positive correlation between GLUL and GPER expression in the TNBC microenvironment. High cytoplasmic GPER expression was associated with adverse pathological features, such as high TNM stage, poor histology grade, and reduced disease‐free survival and overall survival in patients with TNBC. Consistent with in vitro data, mice injected with MDA‐MB‐231 and CAFs/shGPER+shGLUL had the fewest pulmonary metastatic nodules in vivo. Thus, this study revealed that cytoplasmic GPER mediated the biosynthesis and secretion of glutamine via the cAMP/PKA/CREB/GLUL pathway in CAFs through both in vivo and in vitro experiments, further delineating that the unique function of microenvironment GPER signalling has a critical impact on TNBC metabolism to stimulate tumour progression.

ASCT2 and GLS1 are crucial regulators of tumour progression in breast cancer.^29^, ^30^, ^39^ Various factors regulate ASCT2 and GLS1. For example, CD9 enhances the placement of ASCT2 at the plasma membrane, thereby increasing glutamine absorption in pancreatic ductal adenocarcinoma.^40^ Lobetyolin inhibits ASCT2 expression via suppressing c‐Myc.^41^ GLS1 can be regulated by miRNAs (e.g., miR‐145,^42^ miRNA‐192^43^) and signalling pathways (e.g., AKT/GSK3b/cyclinD1).^44^ This study found stromal GPER‐activated CAFs enhance glutamine uptake and metabolism in TNBC cells via ASCT2 and GLS1, supplying carbon and nitrogen to enhance cell proliferation and survival. Interestingly, lactate production from GPER‐activated CAFs increases ASCT2 and GLS1 expression in TNBC cells. Lactate and protons within the tumour microenvironment serve as active mediators rather than mere byproducts, playing critical roles in facilitating tumour progression. Lactate could activate G protein‐coupled receptor GPR81, promoting angiogenesis, immune evasion, and chemoresistance.^45^, ^46^ There may be some possible limitations in this study. First, the precise mechanism by which lactate enhances ASCT2 and GLS1 expression to boost OXPHOS in TNBC cells remains unknown and requires further exploration. Next, the present study only represents the effects of the GPER+ CAFs subgroup (more than 60% population) on TNBC progression and does not involve the biological functions of more detailed CAF subgroups such as matrix CAFs, inflammatory CAFs, etc.^47^, ^48^ Thus, the heterogeneity of GPER+ CAFs will be gradually explored in the future.

In conclusion, estrogen‐activated microenvironmental GPER in CAFs enhances GLUL and LDHB expression via the cAMP/PKA/CREB signalling pathway, facilitating glutamine production and utilization. Glutamine serves as a crucial mediator of metabolic coupling between CAFs and TNBC cells, facilitating tumour cell growth, invasion, metastasis, and drug resistance by enhancing mitochondrial function. Targeting the metabolic coupling between CAFs and TNBC cells triggered by estrogen/cytoplasmic GPER/GLUL signalling in vivo may offer a promising therapeutic strategy to improve clinical outcomes for patients suffering from TNBC.

## AUTHOR CONTRIBUTIONS

Tenghua Yu, Bin Xu, Chunling Jiang, and Yu‐an Qiu contributed to study conceptualization and project administration. Zhengkui Sun, Jiawei Xu, and Manran Liu contributed to data curation and formal analysis. Liyan Liu, Yanxiao Huang, Zhiqiang Peng, and Bin Xu conceived and designed the experiments. Meixi Peng, Xiaoqiang Zeng, and Jiawei Xu contributed to validation and visualization. Chongwu He, Meixi Peng, Xiaoqiang Zeng, and Hanzhi Dong contributed to writing the original draft. Chongwu He, Meixi Peng, Xiaoqiang Zeng, and Tenghua Yu contributed to the revision of the manuscript. Zhengkui Sun and Tenghua Yu contributed to the investigation and methodology. All authors read and approved the final manuscript.

## CONFLICT OF INTEREST STATEMENT

The authors declare no conflict of interest.

## ETHICS STATEMENT

The study received approval from the Ethics Committee of Jiangxi Cancer Hospital, and all participants consented to the study and its publication.

## Supporting information



Supporting Information

Supporting Information

Supporting Information

Supporting Information

Supporting Information

Supporting Information

Supporting Information

Supporting Information

Supporting Information

## Data Availability

All the data are available from the corresponding author Tenghua Yu upon reasonable request.
