# Supplementary figures and images for: Microenvironmental G protein‐coupled estrogen receptor‐mediated glutamine metabolic coupling between cancer‐associated fibroblasts and triple‐negative breast cancer cells governs tumour progression
Source: Clin Transl Med. 2024 Dec 17;14(12):e70131. doi: 10.1002/ctm2.70131 (PMC11652115; doi:10.1002/ctm2.70131)

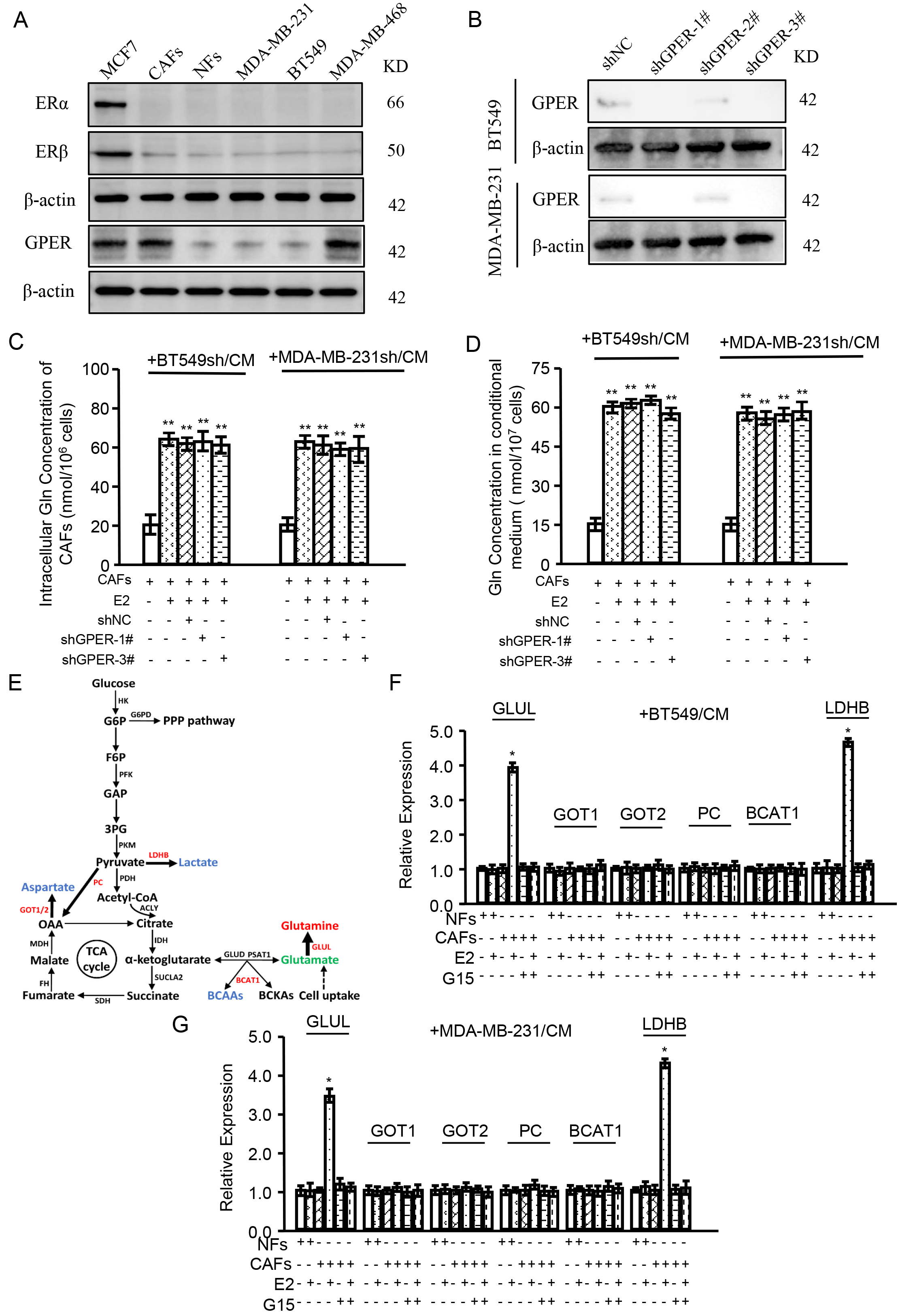

Supplement: Supplementary file 1 — Supporting Information [file CTM2-14-e70131-s003.tif]

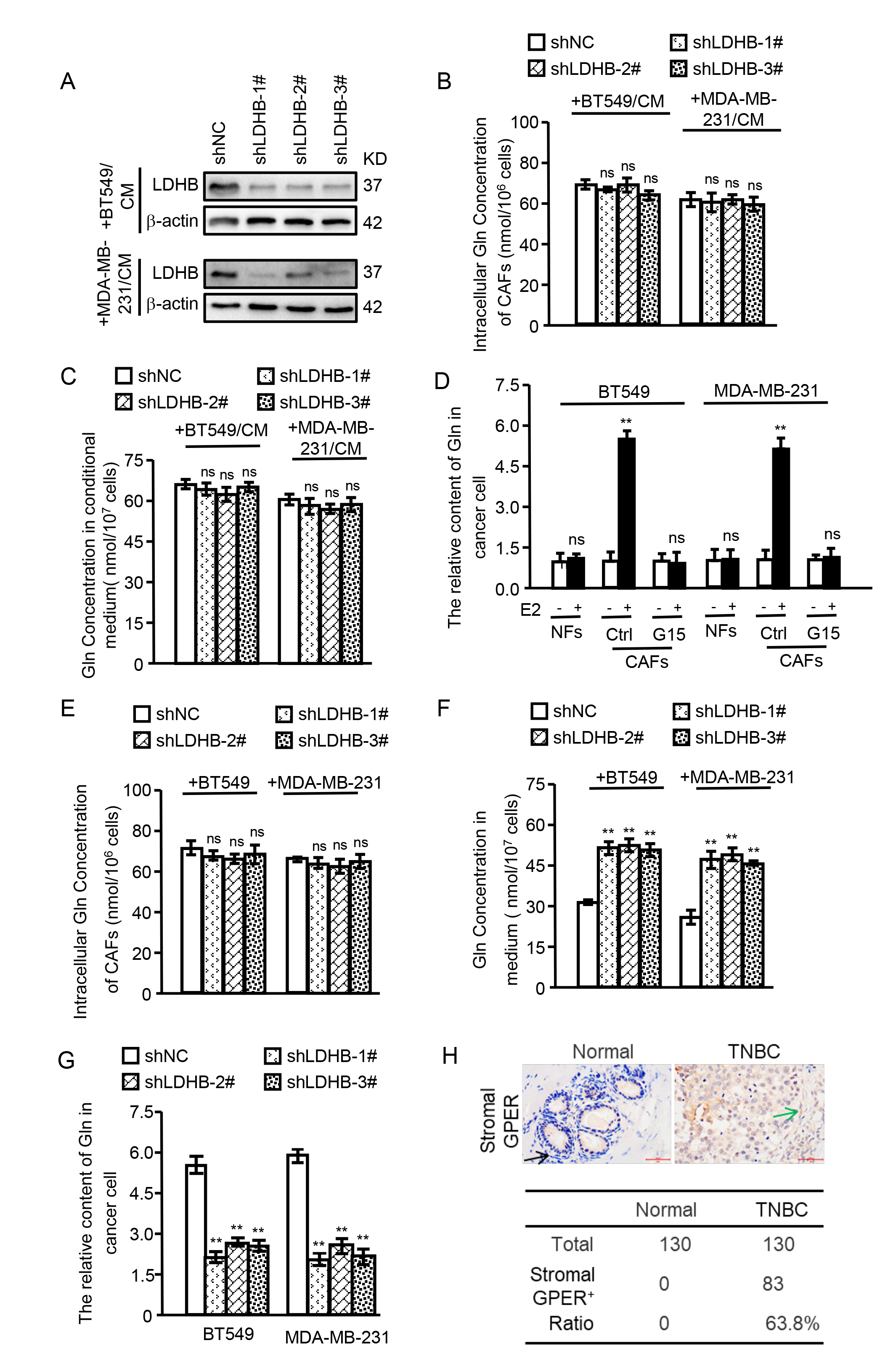

Supplement: Supplementary file 2 — Supporting Information [file CTM2-14-e70131-s004.tif]

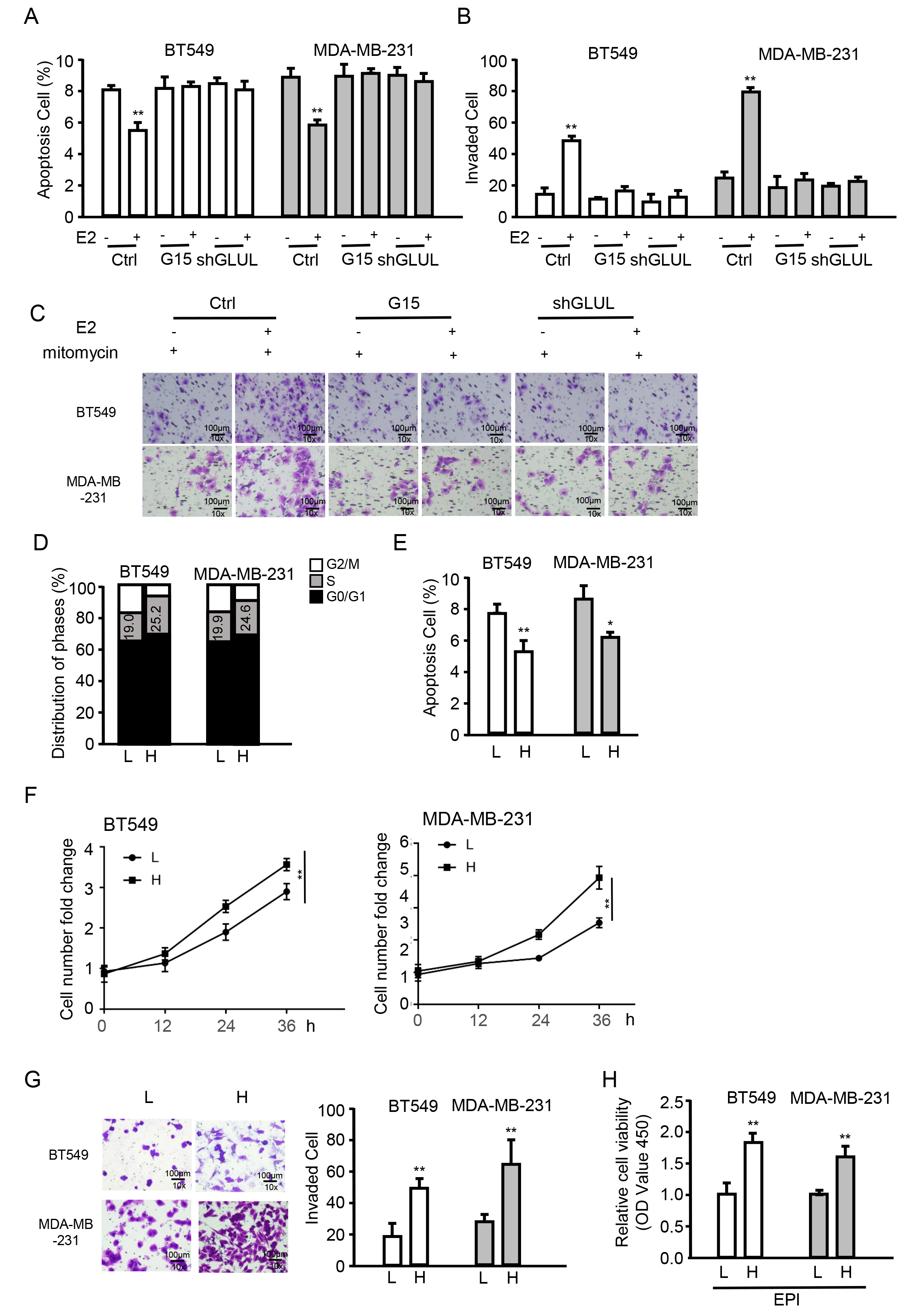

Supplement: Supplementary file 3 — Supporting Information [file CTM2-14-e70131-s006.tif]

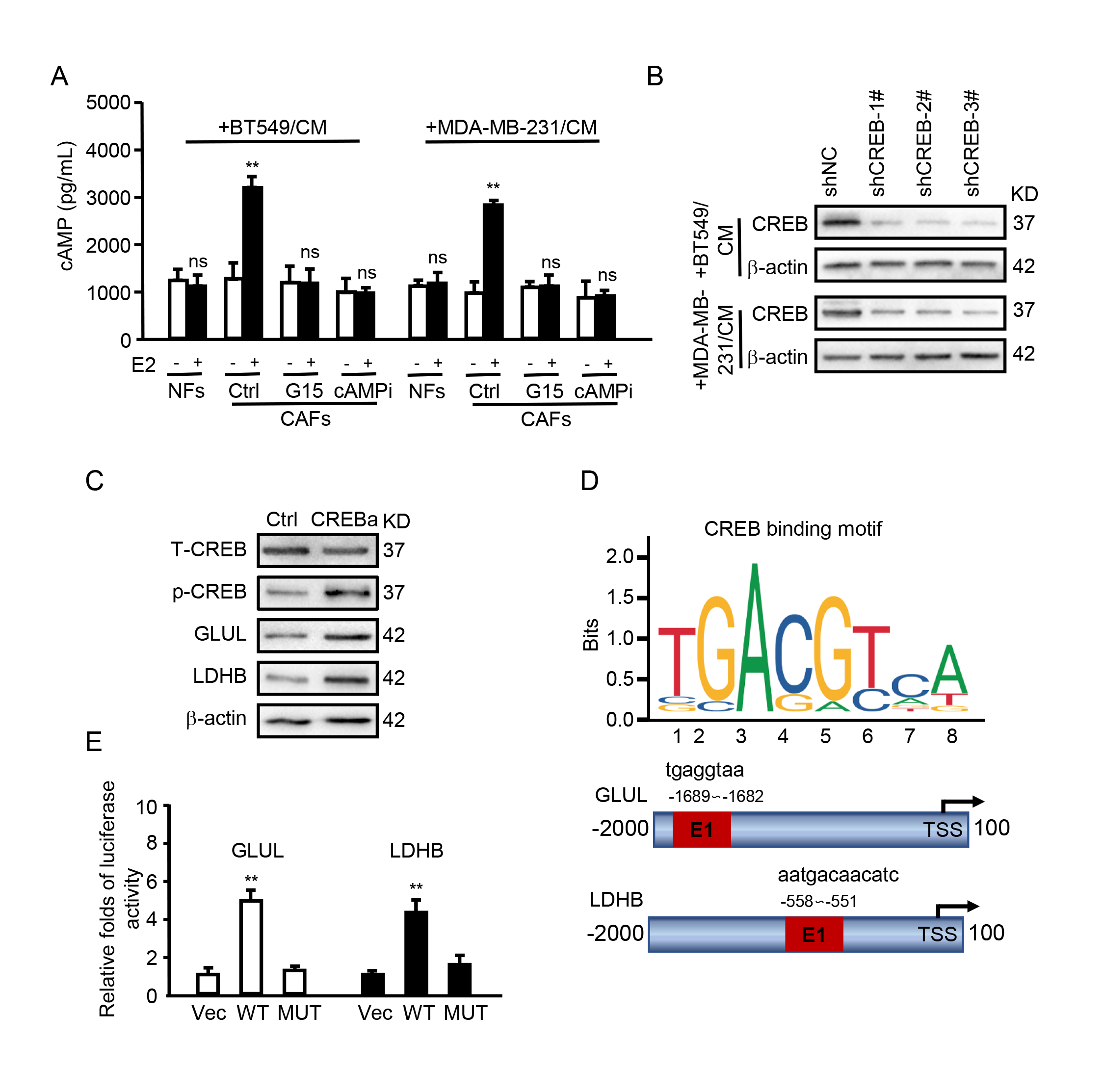

Supplement: Supplementary file 4 — Supporting Information [file CTM2-14-e70131-s007.tif]

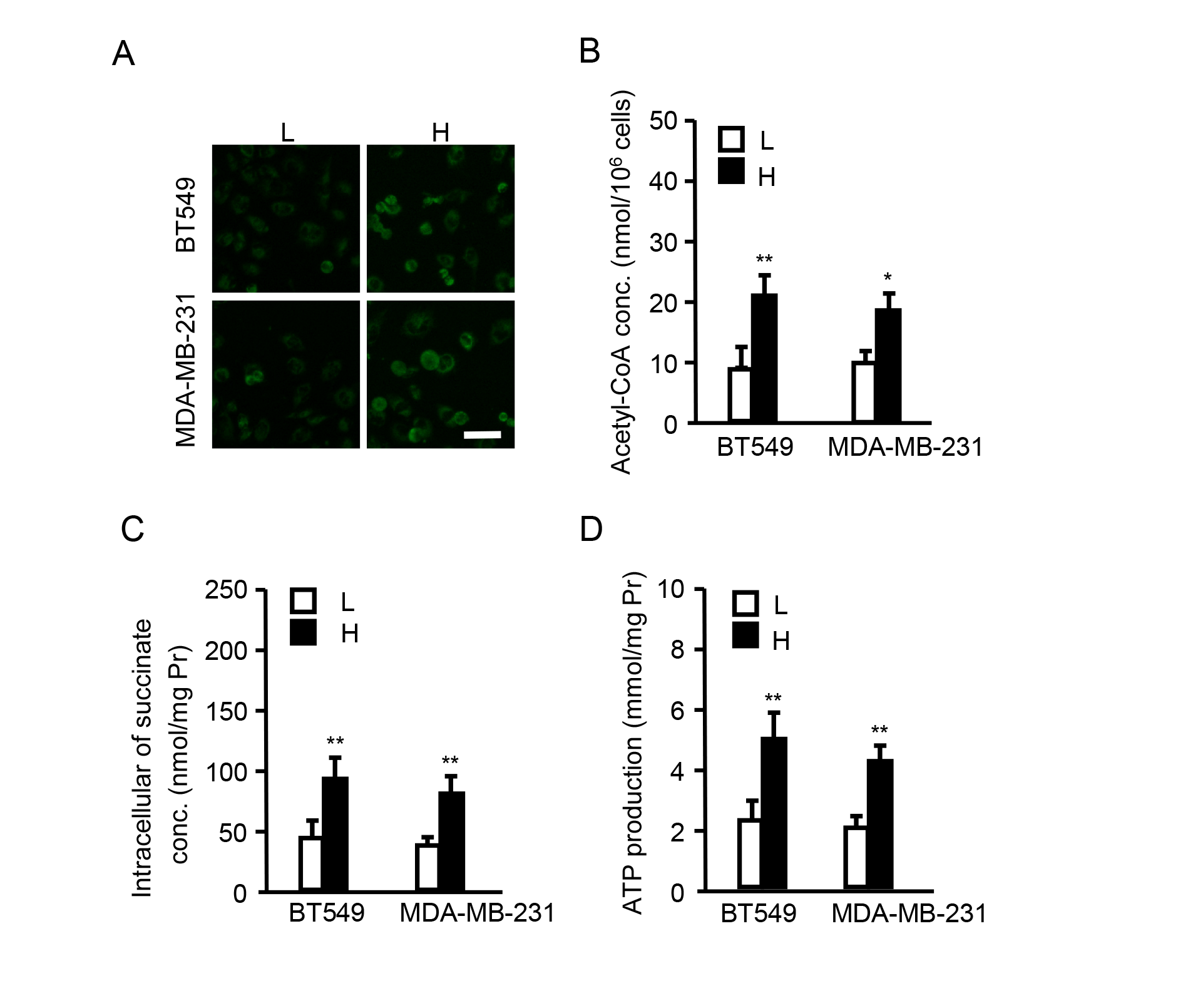

Supplement: Supplementary file 5 — Supporting Information [file CTM2-14-e70131-s009.tif]
