# Supplementary material for: Microenvironmental G protein‐coupled estrogen receptor‐mediated glutamine metabolic coupling between cancer‐associated fibroblasts and triple‐negative breast cancer cells governs tumour progression
Source: Clin Transl Med. 2024 Dec 17;14(12):e70131. doi: 10.1002/ctm2.70131 (PMC11652115; doi:10.1002/ctm2.70131)
Supplement: Supplementary file 6 — Supporting Information [file CTM2-14-e70131-s002.docx]

Supplementary Table 1: The shRNA sequence used in this study.

| Gene Symbol | Sequence name | shRNA sequence |
| --- | --- | --- |
| GPER | shGPER-1# | CGCTCCCTGCAAGCAGTCTTT |
|  | shGPER-2# | CGAGTTAATGAGGAGATGGAA |
|  | shGPER-3# | CGCTCCCTGCAAGCAGTCTTT |
| GLUL | shGLUL-1# | GCACACCTGTAAACGGATAAT |
|  | shGLUL-2# | GCCATGTATATCTGGATCGAT |
|  | shGLUL-3# | GCATCGTGTGTGTGAAGACTT |
| CREB | shCREB-1# | GCAGAACAAATCTCAGCAGAA |
|  | shCREB-2# | GCTGGGAAGACAGATGCTAAT |
|  | shCREB-3# | GAGTTGGTATCCAGTCGAGAA |
| LDHB | shLDHB-1# | CGTGATTGGAAGTGGATGTAA |
|  | shLDHB-2# | GCTTATTTCTTCAGACACCTA |
|  | shLDHB-3# | GCGTTATCAACCAGAAGCTAA |

Supplementary Table 2: The primer used in this study.

| Gene Symbol | Forward (5′-3′) | Reverse (5′-3′) |
| --- | --- | --- |
| GAPDH | TGACTTCAACAGCGACACCCA | CACCCTGTTGCTGTAGCCAAA |
| GPER | CACCAGCAGTACGTGATCGG | CATCTTCTCGCGGAAGCTGAT |
| GLUL | AAGAGTTGCCTGAGTGGAATTTC | AGCTTGTTAGGGTCCTTACGG |
| GOT1 | ATGGCACCTCCGTCAGTCT | AGTCATCCGTGCGATATGCTC |
| GOT2 | AAGAGGGACACCAATAGCAAAAA | GCAGAACGTAAGGCTTTCCAT |
| PC | ACAGAGGTGAGATTGCCATCC | CACTGCATCTACGTTGTTCTCC |
| BCAT1 | GTGGAGTGGTCCTCAGAGTTT | AGCCAGGGTGCAATGACAG |
| LDHB | TGGTATGGCGTGTGCTATCAG | TTGGCGGTCACAGAATAATCTTT |
