# Supplementary material for: Microenvironmental G protein‐coupled estrogen receptor‐mediated glutamine metabolic coupling between cancer‐associated fibroblasts and triple‐negative breast cancer cells governs tumour progression
Source: Clin Transl Med. 2024 Dec 17;14(12):e70131. doi: 10.1002/ctm2.70131 (PMC11652115; doi:10.1002/ctm2.70131)
Supplement: Supplementary file 7 — Supporting Information [file CTM2-14-e70131-s001.docx]

| Gene Symbol | Forward (5′-3′) | Reverse (5′-3′) |
| --- | --- | --- |
| GAPDH | TGACTTCAACAGCGACACCCA | CACCCTGTTGCTGTAGCCAAA |
| GPER | CACCAGCAGTACGTGATCGG | CATCTTCTCGCGGAAGCTGAT |
| GLUL | AAGAGTTGCCTGAGTGGAATTTC | AGCTTGTTAGGGTCCTTACGG |
| GOT1 | ATGGCACCTCCGTCAGTCT | AGTCATCCGTGCGATATGCTC |
| GOT2 | AAGAGGGACACCAATAGCAAAAA | GCAGAACGTAAGGCTTTCCAT |
| PC | ACAGAGGTGAGATTGCCATCC | CACTGCATCTACGTTGTTCTCC |
| BCAT1 | GTGGAGTGGTCCTCAGAGTTT | AGCCAGGGTGCAATGACAG |
| LDHB | TGGTATGGCGTGTGCTATCAG | TTGGCGGTCACAGAATAATCTTT |

**Supplementary Table 2: The primer used in this study.**
