# Supplementary material for: Microenvironmental G protein‐coupled estrogen receptor‐mediated glutamine metabolic coupling between cancer‐associated fibroblasts and triple‐negative breast cancer cells governs tumour progression
Source: Clin Transl Med. 2024 Dec 17;14(12):e70131. doi: 10.1002/ctm2.70131 (PMC11652115; doi:10.1002/ctm2.70131)
Supplement: Supplementary file 8 — Supporting Information [file CTM2-14-e70131-s005.docx]

FIGURE S1 GPER mediates the biosynthesis and secretion of glutamine in CAFs.

(A) Western blotting was used to detect the protein expression levels of ERα, ERβ and GPER in MCF-7, CAFs, NFs, MDA-MB-231, BT549, and MDA-MB-468 cells. (B) The efficiencies of GPER silence in the indicated cells were detected by western blotting. (C-D) The production of glutamine in CAFs (C) and the concentration of glutamine in CM (D) from GPER-knockdown TNBC cells are shown (n=3). (E) Schematic showing the synthetic pathway of glutamine and associated critical genes (red-labeled). (F-G) NFs or CAFs were cultured with CM from BT549 (F) or MDA-MB-231(G), and treated with or without indicated reagents (e.g., E2 (100 nM), G15 (100 nM)) for 12 h (n=3). qRT-PCR was employed to determine the expression of indicated glutamine synthesis-related genes (GLUL: glutamate-ammonia ligase, GOT1: glutamic-oxaloacetic transaminase 1, GOT2: glutamic-oxaloacetic transaminase 2, PC: pyruvate carboxylase, BCAT1: branched chain amino acid transaminase 1, LDHB: lactate dehydrogenase B). Data represent mean ± SD. *p* Values were calculated using a student *t* test. The significance of multiple group comparisons was analysed by one-way ANOVA. * *p*< .05, ** *p*< .01.

FIGURE S2 GPER-induced LDHB had no effect on glutamine production in CAFs, but enhanced the uptake of GPER-related glutamine of TNBC cells.

(A) The efficiencies of LDHB silencing in the indicated cells were detected by Western blotting. (B-C) LDHB-silenced CAFs were cultured with CM from BT549 or MDA-MB-231 in the presence of E2, and the production of glutamine in CAFs (B) and the concentration of glutamine in CM (C) were shown (n=3). (D-F) NFs and CAFs were co-cultured with BT549 or MDA-MB-231 using transwells. (D) The relative concentration of glutamine in BT549 and MDA-MB-231 treated with or without indicated reagents (e.g., E2 (100 nM), G15 (100 nM)) was determined (n=3). (E-F) LDHB-silenced CAFs were co-cultured with BT549 or MDA-MB-231 in the presence of E2, and the production of glutamine in CAFs (E) and the concentration of glutamine in medium (F) were shown (n=3). (G) The concentration of glutamine in cancer cells was detected when co-cultured with CAFs with or without LDHB knockdown (n=3). (H) The cytoplasmic GPER expression of normal tissues and TNBC tissues (black and green arrows represent cytoplasmic GPER-negative and -positive stromal fibroblasts). Data represent mean ± SD. *p* Values were calculated using a student *t* test. The significance of multiple group comparisons was analysed by one-way ANOVA. ** *p*< .01; ns, no significance).

FIGURE S3 Microenvironmental GPER enhances TNBC cells malignant potential *via* glutamine metabolism.

(A) The percentage of apoptosis cell was calculated (n=3). (B) BT549 and MDA-MB-231 were treated with indicated reagents (e.g., E2 (100 nM, 12 h), G15 (100 nM, 12 h)), or subjected to gene knockdown and co-cultured with NFs or CAFs. The invasion of BT549 and MDA-MB-231 cells was evaluated *via* transwell assay, and quantitative diagrams of invaded cells were shown (n=3). (C) BT549 and MDA-MB-231 treated with E2 (100 nM, 12 h), with or without mitomycin (an antiproliferative compound, 25 μg/mL), co-cultured with CAFs respectively. The invasion of BT549 and MDA-MB-231 were evaluated *via* transwell assay. Scale bar, 100 μm(magnification, × 100). (D-H) BT549 and MDA-MB-231 were treated with high (H, 4 mM) and low level (L, 1mM) levels of glutamine. (D) The S-phase cell ratio of BT549 and MDA-MB-231 cells was measured by flow cytometry. (E) Cell apoptosis of BT549 and MDA-MB-231 cells was determined using Annexin V-FITC Kit and ﬂow cytometry, and the percentage of apoptosis cell was calculated (n=3). (F) Cellular proliferation assays were conducted by using CCK8 assay (n=3). (G) The invasion of BT549 and MDA-MB-231 cells was evaluated *via* transwell assay. Scale bar, 100 μm(magnification, × 100). (H) The viable cells of BT549 and MDA-MB-231 cells in the presence of EPI (1.2 μg/ml) were determined by CCK8 kit (n=3). Data represent mean ± SD. *p* Values were calculated using a student *t* test. The significance of multiple group comparisons was analysed by one-way ANOVA. * *p*< .05; ** *p*< .01.

FIGURE S4 GLUL and LDHB are regulated by CREB at the translational level.

(A) NFs or CAFs were cultured with CM from BT549 or MDA-MB-231 and treated with indicated reagents (e.g. E2 (100 nM), G15 (100 nM), MDL-12330 (20 µM)) for 12 h. The production of cAMP in NFs and CAFs was detected using an ELISA Kit (R&D System, Minneapolis, MN, USA) (n=3). (B) The efficiency of CREB silencing in the indicated cells was detected by western blotting. (C) The expression of T-CREB, p-CREB, GLUL, and LDHB in CAFs treated with or without astramembrangenin (20 µM, 12 h) were determined by western blotting. (D) The CREB binding motif (upper) and site (lower) in the GLUL and LDHB gene promoters were shown *via* bioinformatics analysis. (E) Wild-type and mutated GLUL or LDHB promoter luciferase reporter plasmid (RLuc-GULU/LDHB) were transfected into HEK-293T cells with CREB plasmids or control plasmids. Luciferase activity values were measured, analyzed and normalized to the empty vector control (n=3). Data represent mean ± SD. *p* Values were calculated using a student *t* test. The significance of multiple group comparisons was analysed by one-way ANOVA. ** *p*< .01, ns, no significance.

FIGURE S5 Microenvironmental glutamine enhanced mitochondria activity in TNBC cells.

(A-D) BT549 or MDA-MB-231cells were treated with high (4 mM) and low (1 mM) levels of glutamine in the presence of lactate (20 mM). Mitochondria activity (A), acetyl-CoA concentration (B) (n=3), succinate concentration (C) (n=3), and ATP production (D) (n=3) were detected using corresponding reagent kits. Scale bars, 50 μm (magnification, × 400). Data represent mean ± SD. *p* Values were calculated using a student *t* test. * *p*< .05; ** *p*< .01.
