# Supplementary material for: Microenvironmental G protein‐coupled estrogen receptor‐mediated glutamine metabolic coupling between cancer‐associated fibroblasts and triple‐negative breast cancer cells governs tumour progression
Source: Clin Transl Med. 2024 Dec 17;14(12):e70131. doi: 10.1002/ctm2.70131 (PMC11652115; doi:10.1002/ctm2.70131)
Supplement: Supplementary file 9 — Supporting Information [file CTM2-14-e70131-s008.docx]

Supplementary materials and methods

1.1 | Metabolome detection and data analysis

CAFs were treated with or without 17β-estradiol (E2, 100 nM, 12 h). UHPLC-MS/MS analysis was conducted using Vanquish UHPLC system coupled with an Orbitrap Q Exactive series mass spectrometer (Thermo Fisher, American). Metabolites were annotated using the Kyoto Encyclopedia of Genes and Genomes (KEGG) database and the Human Metabolome Database (HMDB). Metabolites with variable importance in projection (VIP) > 1 and *p*-value < 0.05, and fold change ≥ 2 or FC ≤ 0.5 were considered differential metabolites. The functions of these metabolites and metabolic pathways were analyzed using the KEGG database.

1.2 | Cell culture

BT549 was cultured in RPMI-1640 (Gibco-BRL, Australia) supplemented with 10% fetal bovine serum (FBS) (Gibco-BRL, Australia). MDA-MB-231, NFs and CAFs were maintained in Dulbecco’s modified Eagle’s medium (DMEM) (Gibco-BRL, Australia) with 10% FBS. Cells were cultured at 37 °C in a humidified atmosphere containing 5% CO2. Before the experiments, all cells were transferred to glutamine-and phenol-free DMEM or RPMI-1640 with FBS for 24 h, except where noted.

1.3 | Fibroblasts isolation and immortalization

Breast tumor specimens and adjacent normal tissues were washed with PBS containing 100 U/ml penicillin and 100 μg/ml streptomycin (Sigma, St. Louis, MO, USA), then minced into small pieces and digested for 8 h at 37 °C in DMEM supplemented with 10% FBS and 0.5 mg/ml collagenase (Sigma). After centrifugation and washing with PBS, the cell pellets were re-suspended in DMEM with 10% FBS and transferred into 100-mm tissue-culture dishes. CAFs and NFs were routinely maintained in DMEM containing 10% FBS at 37 °C in a humidified atmosphere with 5% CO2.

Fibroblasts were immortalized by hTERT. The retroviral vector pBABE-hygro-hTERT or pBABE-hygro was transfected into the PT67 cell line using Lipofectamine (Invitrogen, Carlsbad, CA, USA). Retroviruses were harvested 48 h after transfection and were employed to infect fibroblasts in the presence of 4 μg/mL polybrene (Sigma).

1.4 | Reagents

The flowing antibodies were used in Western blotting: anti-LDHB (1:1000; Cat. no. ab75167), GLUL (1:1000; Cat. no. ab64613), PC (1:1000; Cat. no. ab220363), BCAT1(1:1000; Cat. no. ab232706), T-PKA (1:1000; Cat. no. ab38949), p-PKA (1:1000; Cat. no. ab32390), T-CREB (1:1000; Cat. no. ab32515), p-CREB (1:1000; Cat. no. ab32096), ASCT2 (1:1000; Cat. no. ab104921) and GLS1 (1:1000; Cat. no. ab260047) were purchased from Abcam (Cambridge, MA, USA). Anti-GOT1 (1:1000; Cat. no. 34423s) and β-actin (1:1000; Cat. no. 3700) were obtained from Cell Signaling Technology (Shanghai, China), and GOT2 (1:500; Cat. no. MB9242) was purchased from Bioworld (St. Louis Park, MN, USA). The flowing antibodies were used in IHC: anti-GPER (1:200; Cat. no. ab39742), α-SMA (1:200; Cat.no. ab265588), GLUL (1:200; Cat. no. ab64613) and GLS1 (1:200; Cat. no. ab260047) were purchased from Abcam (Cambridge, MA, USA).

1.5 | Preparation of conditioned medium (CM)

BT549, MDA-MB-231, CAFs, or NFs were cultured in a growth medium with 10% FBS to approximately 90% confluence. The growth medium was then replaced with glutamine-and phenol-free DMEM or RPMI-1640 with 1% FBS medium, and the cells were further cultured for 30 hours. The supernatant was collected as a conditional medium (CM). Thus, the CM of BT549 was derived from glutamine-and phenol-free RPMI-1640 medium, whereas the CM of glutamine-and phenol-free MDA-MB-231, NFs, and CAFs were derived from the DMEM medium.

1.6 | Lentiviral vectors and cell infection

The lentivirus-based short hairpin RNA (shRNA) vectors targeting GPER, GLUL, CREB, and LDHB and scramble lentiviral vectors were purchased from Genechem (Shanghai, China). The indicated cells were infected with lentivirus for 48 h in the presence of 5 μg/mL polybrene (Sigma, CA, USA), after which they were subjected to 2 μg/mL puromycin selection for 7 d (Sigma). The puromycin-resistant cells were isolated and propagated for further analysis. The shRNA sequences are listed in Supplementary Table 1.

1.7 | Western blotting

Western blotting analysis was conducted as previously described 23. Briefly, total cell proteins were extracted using RIPA lysis buffer (Beyotime, China), quantified with BCA protein assay kit (Beyotime, China), resolved in a 10% SDS-PAGE gel, and incubated with the specified primary antibodies. Horseradish peroxidase-conjugated anti-mouse or anti-rabbit IgG antibody (ZSGBBIO, China) served as a second antibody. Protein bands were visualized using the enhanced chemiluminescence system (Amersham Pharmacia Biotech, Tokyo, Japan).

1.8 | Reverse transcription and real-time PCR

Total RNA was extracted using TRIzol^®^ (Invitrogen, Carlsbad, CA, USA) according to the manufacturer's protocol. Reverse transcription was performed using the PrimeScript RT reagent kit (Takara, Dalian, China). Real-time PCR was conducted with SYBR Premix Ex TaqTM II (Takara). In the drug treatment groups, cells were treated with E2 (100 nM), G15 (100 nM), MDL-12330 (20 µM), H-89 (30 μM), EPI (1.2 μg/ml) at the specified time points as described in the ﬁgure legends and then tested for mRNA expression of genes. Gene expression was calculated using the comparative 2−^ΔΔ^CT method with GAPDH used as the reference standard. The gene primers are listed in Supplementary Table 2. All experiments were performed at least three times.

1.9 | Luciferase reporter assay

To confirm that CREB binds to the GLUL/LDHB promoter, the full-length CREB cDNA was cloned into the pcDNA3.1 vector to construct the CREB-expressing plasmid pcDNA3.1-CREB. Wild-type or mutant regions 2000 bp upstream of the GLUL/LDHB promoter (identified by JASPAR) were cloned into the pGL3 plasmid (Promega) to construct pGL3-GLUL/LDHB reporter plasmids. Transfection was performed using Lipofectamine 3000 with these plasmids and Renilla luciferase reporter PRL-TK plasmids. After 48 h, the Dual-Luciferase Reporter Assay System was employed to measure luciferase activity, normalizing transfection efficiencies to Renilla activity. The dual-luciferase activity of the transfected 293T cells was measured using a Thermomax microplate reader.

1.10 | Measurement of glutamine concentration, lactate production, ATP production, acetyl-CoA concentration, succinate concentration and glucose consumption

In the co-culture system, cells treated with indicated reagents were analyzed for glutamine concentration, lactate production, ATP production, acetyl-CoA concentration, succinate concentration and glucose consumption using the following kits: glutamine and glutamate determination kit (Sigma, GLN1-1KT), LA assay kit (Solarbio® BC2230), ATP content assay kit (Solarbio® BC0300), acetyl-CoA assay kit (Solarbio® BC0980), succinate colorimetric assay kit (Sigma® MAK184), and glucose assay kit (Solarbio® BC2500), according to the manufacturers' instructions. respectively. All experiments were performed at least three times, and the data were normalized by cell numbers or protein content.

1.11 | Cell invasion assays

To negate the effects of cell proliferation on cell invasion, cancer cells were treated with 25 μg/mL mitomycin. A modified Boyden chamber assay was used for cell invasion assays as described previously24. Brief, 3 × 104 cancer cells in 200 μl of FBS-free medium were seeded in the wells of 8 μm-pore Boyden chamber (Millipore Darmstadt, Germany) and cultured for 48h. After removing the Matrigel from the chamber with a cotton applicator, the invaded cells on the opposed filter were stained with hematoxylin in methanol and counted in five of the randomly selected visible fields. All experiments were repeated at least three times.

1.12 | CCK-8 assays

A total of 3 × 103 cells were seeded into each well of a 96-well plate in 100 μL of growth medium. After culturing in the phenol-free medium for 24 h, cells were treated with either E2 (100 nM), G15 (100 nM), or these drugs combined with MDL-12330 (20 µM), H-89 (30 μM), or EPI (1.2 μg/mL). Five replicate wells were used for each treatment. After incubating for 24 h, 10μL of CCK-8 (APExBIO, K1018) was added to each well, followed by a 2-hour incubation. Absorbance was recorded using an ultraviolet spectrophotometric reader at a wavelength of 450 nm.

1.13 | Flow cytometric analysis

In the transwell co-culture system between CAFs and breast cancer cells, breast cancer cells in the S-phase of the cell cycle were identified using standard propidium iodide staining, while apoptotic cells were detected using standard the Annexin V-FITC and Propidium Iodide Kit (Beyotime, China). Flow cytometry analysis was conducted with a Flow cytometer (BD FACSAria™ Fusion, BD, American). All experiments were performed in triplicate.
